# Integrating Physical Principles with Machine Learning for Predicting Field-Enhanced Catalysis

**DOI:** 10.1021/jacsau.4c00901

**Published:** 2025-02-17

**Authors:** Runze Zhao, Qiang Li, Jiaqi Yang, Cheng Zhu, Fanglin Che

**Affiliations:** † Department of Chemical Engineering, University of Massachusetts Lowell, Lowell, Massachusetts 01854, United States; ‡ Engineering Directorate, Lawrence Livermore National Laboratory, 7000 East Avenue, Livermore, California 94550, United States

**Keywords:** density functional theory, machine learning, local electric field mapping, vibrational stark effect, catalyst nanoparticles

## Abstract

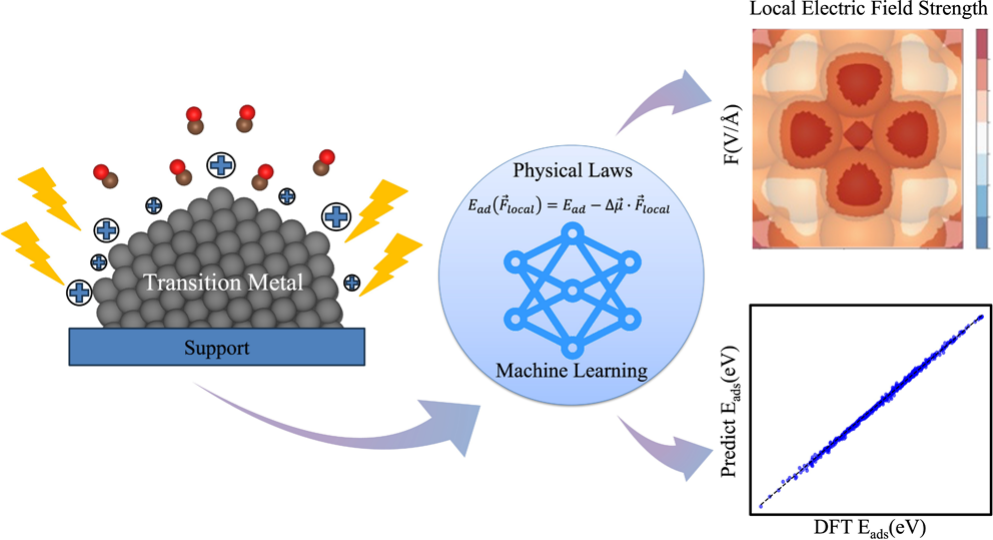

Field–dipole interactions can tune the energetics of polarized species over catalyst nanoparticles (NPs) for sustainable technologies. This can boost the energy efficiency of desired reactions by several orders of magnitude compared with conventional heating. However, the local electric field accumulation over the NPs sharp points and field-dependent adsorption over NPs are not well studied, and the associated computational expense is immense. To address this challenge, we introduce an innovative approach that combines density functional theory (DFT) calculations, DFT-based CO vibrational Stark effects, and physics principles enhanced machine learning (ML). This approach enables precise mapping of local electric fields and integrates the physical principles of the first-order Taylor expansion as a training input into the ML model for predicting field-dependent adsorption, facilitating rapid prediction of field-dependent adsorption energetics with acceptable accuracies, particularly when training data sets are limited. Our methodology reveals the dominant roles of external electric field (EEF), the generalized coordination number (GCN), and NP size in determining the local electric field (LEF) strength. Low-coordinated sites and small NPs size enhanced the LEF by about 4-fold compared to the flat surfaces. Using ML models, we can predict the field-driven adsorption energetics at a given adsorption site of the NPs with high accuracy and efficiency. The integration of *ab initio* modeling and ML algorithms offers exceptional possibilities to facilitate catalyst development and create the opportunity to enter a new paradigm in field-enhanced catalysis design based on fundamentals rather than trial and error.

## 1 Introduction

Electric fields can rearrange the molecular orbitals, tune the energetics of polarized species over the catalysts, alter the reaction mechanisms, and enhance the catalytic activity by a few orders of magnitude compared to thermal heating. For instance, a local electric field of approximately 0.12 V/Å near cathode, generated by introducing alkali cations into a highly acidic medium, can significantly enhance the CO_2_ electroreduction process, achieving a Faradaic efficiency of up to 90% for the conversion to CO and formic acid.^1−4^ In ammonia synthesis,^5−7^ applying a positive external electric field of 1 V/Å over Ru(0001) can change the reaction rate-limiting step from N_2_* dissociation to N_2_* association with H*.^7^ For methane activation in a fuel cell,^8−12^ a positive external electric field of 1 V/Å can increase methane dissociative adsorption, reduce temperature requirement, and reduce coke formation of 30% over Ni(111). Thus, advanced fundamental science of field-enhanced catalysis can accelerate the discovery of next-generation catalysts for sustainable technologies where large electric field exists, such as electrostatic catalysis,^1−3,13^ plasma catalysis,^14,15^ programmed catalysis,^16,17^microwave catalysis,^18,19^ electrode/electrolyte interfacial kinetics,^20^ and fuel cells.^21,22^

To advance electric-field-enhanced catalysis, one fundamental issue is to precisely measure the local electric field over the exposed catalyst sites under electric conditions. Experimentally, Kelvin probe force microscopy (KPFM)^23^ can probe the local electric field (LEF) under operando conditions by detecting the electrostatic force between a conductive tip and the sample surface, allowing it to map the work function and surface potential with nanoscale resolution. However, traditional KPFM often faces limitations in spatial resolution and encounters challenges in separating the electrostatic signal from topographical features. Typical spatial resolutions for KPFM are in the range of 30–100 nm,^24^ while its ability to accurately measure surface potentials can be affected by tip–sample interactions and reaction environmental factors.^25^ In addition, by monitoring the shift in the probe molecule (e.g., CO) vibrational frequency in *in situ* Infrared spectroscopy, the vibrational stark effect (VSE) allows for the direct quantification of the LEF magnitude at the adsorption site.^26,27^ While this approach is limited to systems where the probe molecule can adsorb, it may not provide a complete picture of the LEF distribution across the entire catalyst surface.

To overcome the experimental limitations of KPFM and Infrared spectroscopy, density functional theory (DFT) calculations can predict the LEF from first principle and estimate the performance of field-enhanced catalysis. Most electric field DFT studies for heterogeneous catalysis are conducted on flat surfaces (e.g., 111), leading to the prediction that an external electric field of over 0.1 V/Å is required to influence catalytic performance. However, for catalyst nanoparticles (NPs), the charges accumulate at low-coordinated points (i.e., defects, corners, edges),^1,28−34^ leading to a much stronger LEF than the external electric field (EEF). Thus, fields somewhat weaker than what is commonly anticipated could be significant. However, the sharp point effects from metallic NPs with tunable shapes, and defects on LEF accumulation^35,36^ and adsorption are not well studied. The associated computational expense is immense.^12,37^

In this work, we present a novel approach that combines density functional theory (DFT) calculations, the DFT-calculated CO vibrational Stark effect (VSE), and machine learning (ML) to accurately map local electric fields (LEFs) and rapidly predict field-dependent adsorption energetics over catalyst NPs. Using CO adsorption on Ni NPs as a model system, we perform DFT calculations on various Ni catalyst models, including slabs and NPs with tunable sizes and shapes, under a range of external electric fields (EEFs, −0.5 to 0.5 V/Å). The LEFs are quickly mapped by the electrostatic potential difference (PD) method and further corrected using DFT-calculated Vibrational Stark shifts. Our results show that the LEF strength is strongly influenced by EEF, the generalized coordination number (GCN), and cluster size. We then developed ML algorithms to reduce *ab initio* calculation costs and fast predicted LEF with a given catalyst NP and an EEF.

We then incorporated physics principles as training inputs for the ML model to improve the prediction accuracy for field-dependent adsorption energetics. The physics principles enhanced ML model can reduce the computational cost of preparing training data sets and deepen the understanding of structure–reactivity relationships in heterogeneous catalysts under electric fields. This approach could pave the way for a new paradigm in field-enhanced catalysis design, driven by fundamental principles rather than traditional trial-and-error methods, and advance renewable energy applications.

## 2 Results and Discussion

### 2.1 Mapping the Local Electric Fields

**Figure 1:**
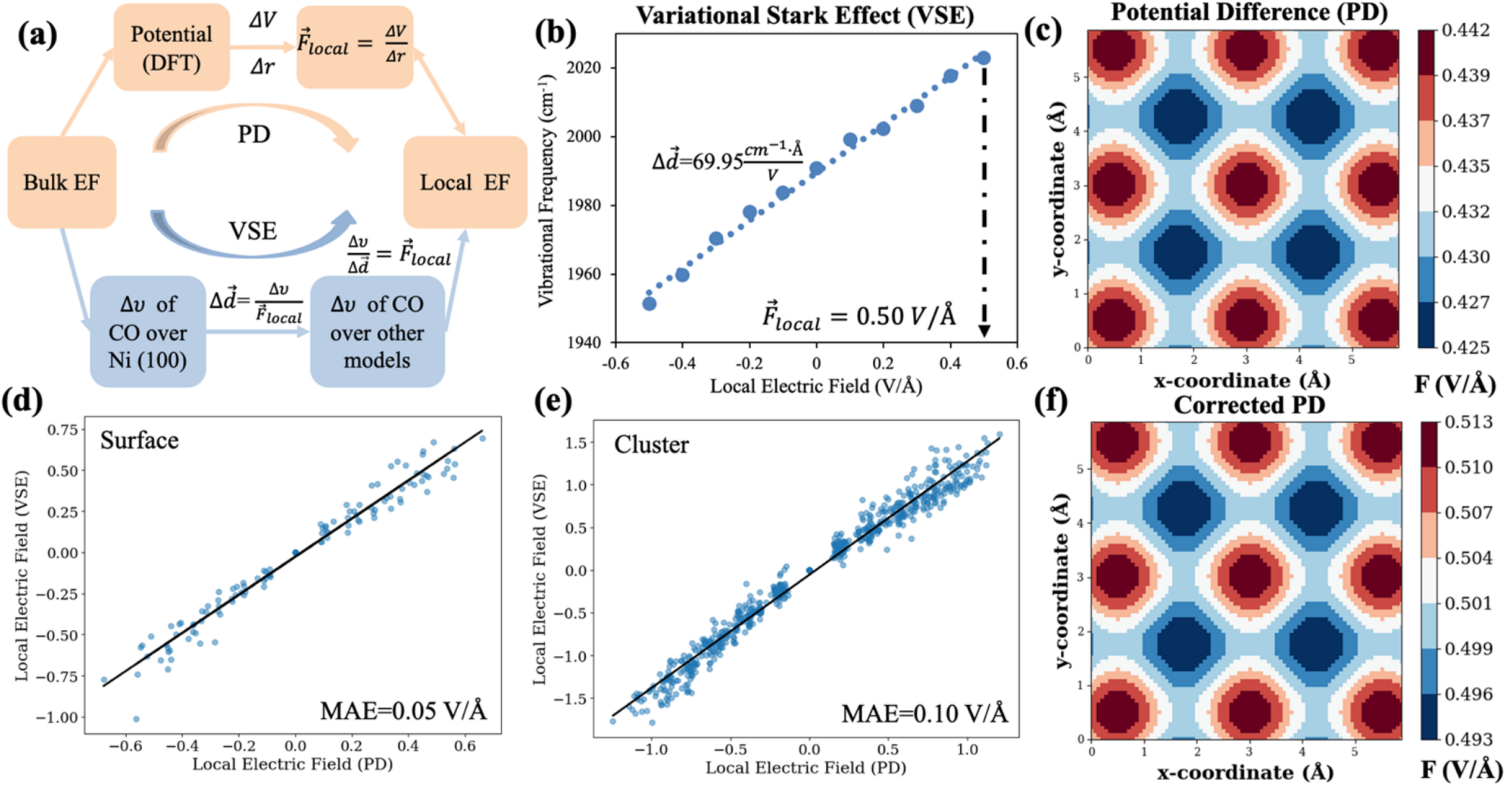
Mapping local electric fields. (a) Schematic illustration of the electrostatic potential difference (PD) and DFT-calculated vibrational stark effect (VSE) methods for the LEF measurement. (b) LEF of the top site of Ni(100) using the VSE method. (c) LEF distribution of the *x*–*y* plane of the Ni(100) surface using the PD method. Relationship between LEF strengths measured by PD and DFT-calculated VSE over slab surfaces (d) and over clusters (e). (f) PD-mapped LEF of the top of Ni(100), via VSE corrections.

Due to the charges accumulating at low-coordinated points (i.e., defects, corners, edges) when an external electric field (EEF) is applied, the LEF distribution over catalyst nanoparticles (NPs) becomes highly heterogeneous. To map the LEF over Ni surfaces and NPs with distinct shapes and sizes (Figures S1–S4), we applied EEF ranging from −0.5 to 0.5 V/Å with an increment of 0.1 V/Å.^38^ CO was adsorbed on top sites of these structures (Figures S5–S10). To quantify the LEF at each adsorbate site, we employed two approaches, including the DFT-calculated vibrational stark effect (VSE) method and the electrostatic potential difference (PD), as illustrated in Figure 1a.

For VSE, the Stark tuning rate (i.e., the dipole moment of the adsorbed CO molecule), which relates the change in the vibrational frequency of the probe molecule (e.g., CO) to the EEF,^26^ was calculated using the following equation:
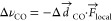
1where Δν_CO_ is the change in the DFT-calculated CO vibrational frequency, Δ*d⃗*_CO_ is the stark tunning rate of CO upon adsorption, and *F⃗*_local_ is the LEF at the adsorption site (Figure 1b). Our implementation of the VSE method relies on two key assumptions. First, we assumed that the LEFs of the top site of Ni(100) are identical to the EEFs. Second, we assumed that the tuning rate (i.e., effective dipole moment of adsorbed CO) remains consistent across different surface environments when it is bound to a single Ni atom in the top configuration.

In the PD method, we calculated the LEF by examining the electrostatic potential difference at the adsorbate center of mass (*r*_COM_). The LEF was then obtained by taking the negative gradient of the electrostatic potential difference. Specifically, for an adsorbed CO molecule:
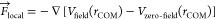
2Here, *V*_field_(*r*_COM_) and *V*_zero-field_(*r*_COM_) are the DFT-computed electrostatic potentials with and without EEF, respectively (Figure 1c). This method provides a straightforward way to map the LEF distribution across all of the surface sites.^3,39^ To ensure that the LEF arises solely from the EEF but not from a combination of geometry changes, we keep atomic positions fixed across calculations with and without EEF when obtaining local potential information. However, this method could depend on the chosen exchange-correlation functional when calculating the electrostatic potentials and has a challenge when compared with the experimental results.

To correct the LEFs mapped by the PD method, we use DFT-calculated vibrational stark effects (VSE) of CO.^27,40^ For the VSE, in the presence of an EEF, a polarized molecule (such as CO) can experience vibrational frequency shifts. Although the VSE of CO is determined through DFT calculations, it is grounded in prior experimental validation studies that show strong agreement between DFT-calculated and experimentally measured VSE (more details are given in Section S2).^27,40−43^ This provides confidence for using DFT-calculated VSE to correct our PD-mapped LEFs.

We then corrected the LEF from the PD method through DFT-calculated VSE method^27,40−43^ (Figure 1d,e). Our database comprises 790 data points derived from DFT calculations, including 121 data points from slab surfaces and 669 data points from cluster models, each containing PD- and VSE-derived LEFs under varying EEFs. The details of this database construction are illustrated in Section S4.2. We evaluated the linear regression (LR) and polynomial regression (PR) models to correlate the LEFs from PD and DFT-calculated VSE methods. While PR and LR exhibit similar performance (Tables S1 and S2), we chose the LR model for its simplicity: For slab surfaces, we found VSE = −0.03 + 1.16 × PD with the mean absolute error (MAE) of 0.05 V/Å, while for cluster surfaces, VSE = −0.06 + 1.33 × PD with an MAE of 0.10 V/Å. Notably, the LEFs of clusters exhibited different correlations between the VSE and PD methods compared to the scenarios of slab surfaces. This difference highlights the importance of considering catalyst geometries, such as the size of NPs, when estimating the local electric fields, especially for catalyst nanoparticles. Figure 1f presents the LR-corrected LEF distribution of the *x*–*y* plane of the Ni(100) surface. At an EEF of 0.5 V/Å, the corrected LEF at the top site is 0.51 V/Å, which also validates our assumption of the DFT-calculated VSE method. With the LR correction implemented, the LEF calculated from the PD method now achieves comparable accuracy as the VSE method and enables mapping of LEFs across all coordinates with less computational expense.

### 2.2 Features Impact the Local Electric Fields

It is practically nontrivial to screen the LEFs across a large search space of NPs, such as various combinations of shapes, sizes, and defects (e.g., adatom, vacancy). To probe the LEF of a given metallic NP catalyst and locate where the maximum LEF could occur, here, we correlate the features, such as EEF, size, and local atomic structure (with generalized coordination number (GCN), being one of the useful descriptors) with the LEF distribution.^1,33,36^

**Figure 2:**
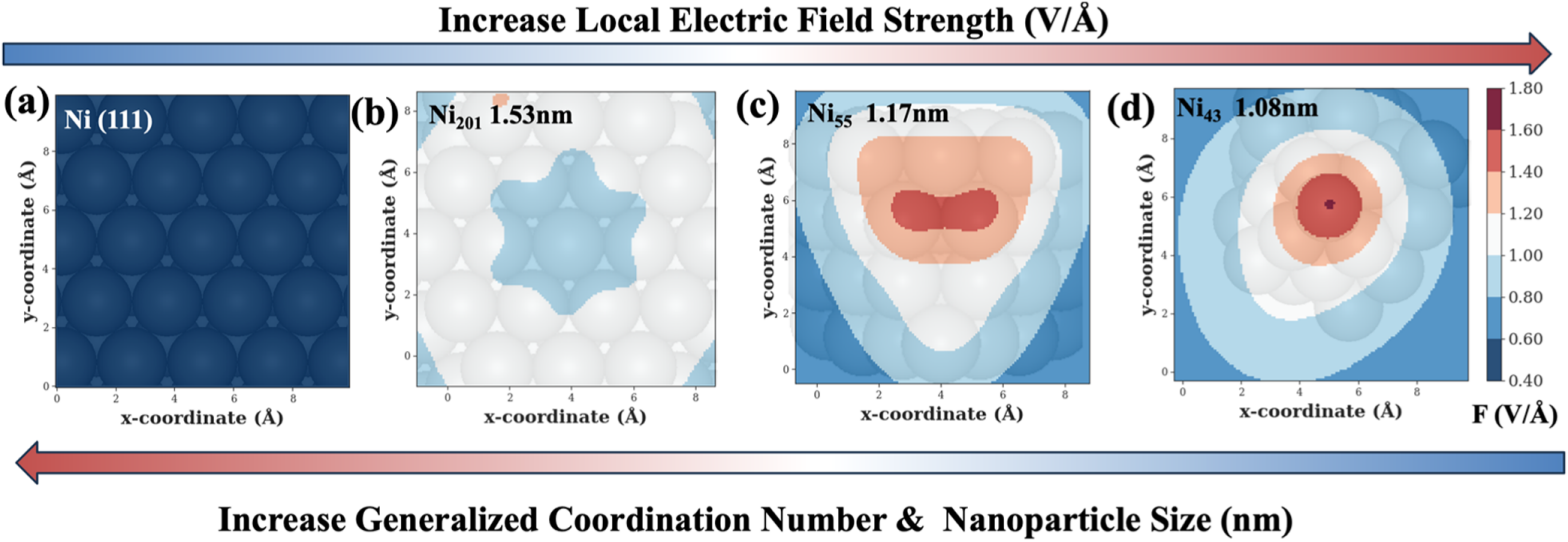
Influence of NP sizes and GCN on local electric fields (LEFs) under certain EEF condition. Under an EEF of 0.5 V/Å, the 2-D *x*–*y* plane map of LEF distribution over the (a) Ni(111) slab (extended surface model, GCN: 7.5), (b) Ni_201_ NP (1.53 nm, GCN: 7.5), (c) Ni_55_ NP (1.17 nm, GCN: 3.92), and (d) Ni_43_ NP (1.08 nm, GCN: 3.08).

Figure 2 depicts the LEF distribution under an EEF of 0.5 V/Å across different surface structures (e.g., extended surfaces can represent large NPs) and small NP cluster sizes. The examined structures include the Ni(111) slab (representing large sizes of NPs, such as >3 nm), and Ni NPs with different sizes and shapes (e.g., 1 and 1.5 nm). The Ni (111) slab with GCN of 7.5 exhibits the lowest LEF, with a value of approximately 0.45 V/Å of the Ni top sites. In addition, the tip of the Ni_43_ cluster (1 nm) with a GCN of 3.08, the tip of Ni_55_ cluster (1.17 nm) with a GCN of 3.92, and the tip of Ni_201_ cluster (1.5 nm) with a GCN of 7.5 experience the LEF of about 1.7, 1.4, and 0.9 V/Å, which are roughly 4, 3.1, and 2 times larger than that of the Ni(111) slab model, respectively. This suggests that as the NP size or GCN increases, the LEF decreases. This phenomenon can be attributed to the differential charge density distribution in the Ni NPs and slabs. Under an EEF, charge tends to accumulate at the tips, corners, and edges, resulting in a higher LEF at these localized spots compared to the more uniform extended slabs (Figure S11). These findings highlight the critical influence of both cluster size and GCN on the magnitude of the LEF under a certain EEF condition.

**Figure 3:**
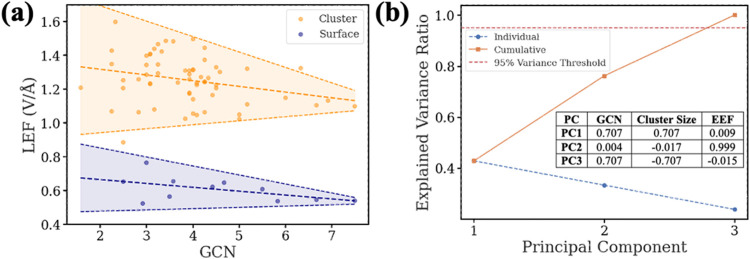
Correlations between LEFs and catalyst model geometries. (a) Relationship between the LEF and GCN for NPs (orange) and surfaces (blue). (b) Explained variance ratio vs principal component plot from PCA. Inset table: Principal component analysis (PCA) loadings for GCN, cluster size, and EEF; orange line: cumulative variance; blue line: individual variance for each component; red dashed line: 95% variance threshold for cumulative variance.

At a specific EEF, the GCN and LEFs are strongly correlated in the slab models. However, in cluster models, the GCN alone does not fully capture the LEFs (Figure 3a). Thus, we further performed a principal component analysis (PCA, Figure 3b)^44^ to assess the roles of EEF, GCN, and cluster size in influencing the LEF predictions. The first three principal components explain 95% of the total variance, validating their necessity. PC1, which primarily captures variations from GCN and cluster size, accounts for the highest variance contribution (∼45%). PC2, which is almost entirely dominated by the EEF (loading: 0.999), reflects the strong effect of EEFs on predicting LEFs. PC3 describes the inverse relationship between the GCN and cluster size. Together, we conclude that EEF, GCN, and size significantly influence the prediction of LEFs.

### 2.3 ML Framework for Predicting LEFs and Field-Dependent Adsorption Energetics

**Figure 4:**
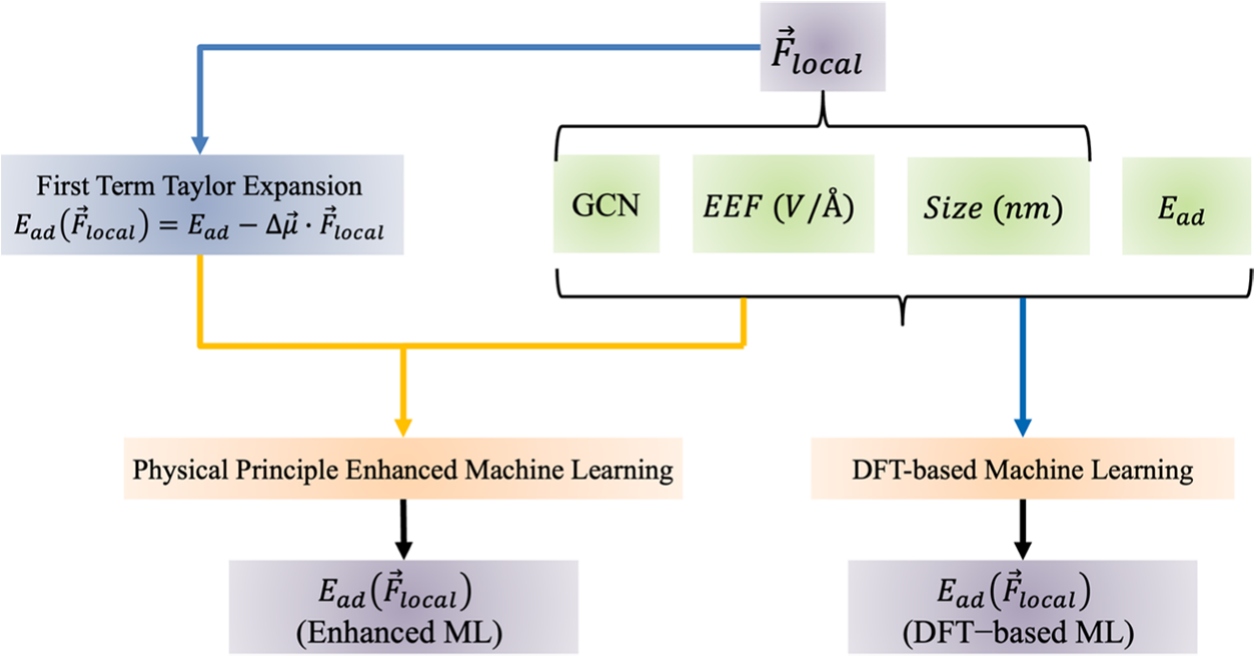
Scheme of the DFT-based ML and physics principles enhanced ML framework for predicting field-dependent CO adsorption energies.

To enhance computational efficiency and maintain acceptable accuracy, we incorporate the physical principles as training inputs into the ML model and develop the physical principles enhanced ML framework, as illustrated in Figure 4. First, an ML algorithm was developed to predict the LEF (V/Å) at catalytic sites using the GCN, cluster size(nm), and EEF (V/Å) as input features. The ML-predicted LEFs were incorporated into the first-order Taylor expansion equation (*E*_ad_(*F⃗*_local_) = *E*_ad_ – Δ*μ⃗*·*F⃗*_local_, where *E*_ad_ represents zero-field CO adsorption energetics, Δ*μ⃗* denotes the effective dipole moment, and *F⃗*_local_ is the LEF predicted from Section 2.3.1) for estimating CO field-dependent adsorption energies. The physics-enhanced ML model predicts field-dependent CO adsorption energetics using input features, such as zero-field CO adsorption energetics, EEFs, GCN, cluster size, and adsorption energetics estimated via first-order Taylor expansion. For comparison, a DFT-based ML model (Section 2.3.2) was also developed to evaluate computational efficiency and accuracy. The key distinction between the physics-enhanced ML model (Section 2.3.3) and the DFT-based ML model lies in the inclusion of Taylor expansion-derived features as training inputs in the former. Further details on the assembly of the ML database and the computational infrastructure employed are provided in Section S4.2 (Figures S12–S15) and Section S4.3 (Tables S4 and S5).

#### 2.3.1 Local Electric Field Prediction

**Figure 5:**
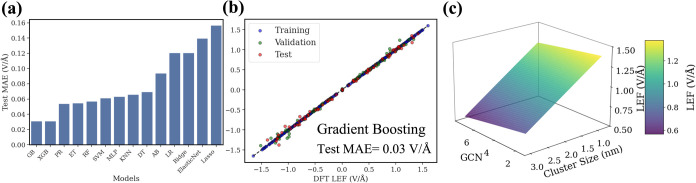
Machine learning performance for local electric field prediction. (a) Test MAE for various ML models, including gradient boosting (GB), XGBoost (XGB), extra tree regressor (ET), random forest (RF), polynomial regression (PR), support vector regression (SVM), multilayer perceptron (MLP), k-nearest neighbors (KNN), decision tree (DT), AdaBoost (AB), linear regression (LR), and ridge regression (Ridge). (b) Comparison of the GB-predicted and DFT-calculated LEF. (c) 3D plot of the LEF distribution as a function of the NP GCN and size at an EEF of 0.5 V/Å.

To predict LEF using the ML algorithm, we divided the data set (790 data points) into 80% for training, 10% for validation, and 10% for testing. A learning curve analysis was performed to ensure an appropriate data selection process (Figure S16). The input features included the GCN, cluster size, and EEF, while the output was the LEF. We compared the performance using the test set MAE for various ML models (Figure 5a). To ensure robust performance estimation and minimize overfitting, we employed 10-fold cross-validation across all models. Hyperparameter optimization was conducted using both grid search and random search methods within the cross-validation framework, which are shown in Table S6.

Among all examined ML algorithms, the GB model demonstrates the best performance and achieves a test set MAE of 0.03 V/Å for predicting the LEF (Figure 5b). Our ML algorithm also revealed a polynomial relationship among GCN, cluster size, and LEFs (Figure 5c). The model predicts that the LEF strength increases as the cluster size and GCN decrease, with the highest LEF strengths occurring at the smallest cluster sizes and lowest GCN values investigated. This trend aligns with our findings from the DFT calculations, where low-coordinated sites and smaller NP exhibited enhanced LEF compared to larger clusters and higher-coordinated surfaces. The ML model’s ability to capture this relationship highlights its potential for accurately predicting LEF distributions based on key structural parameters, providing a computationally efficient alternative to extensive DFT calculations.

**Figure 6:**
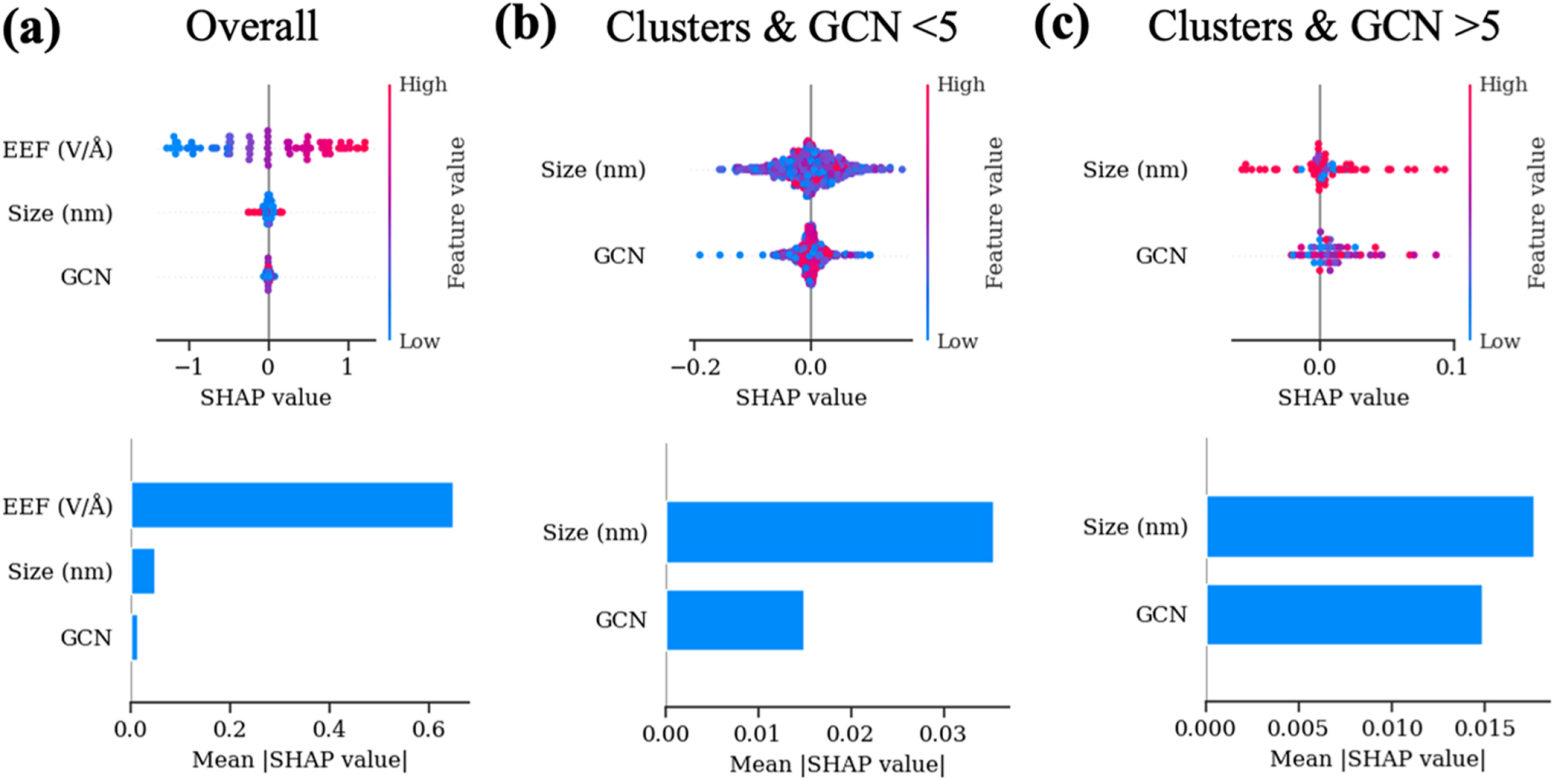
SHAP analysis for LEF predictions. (a) SHAP plot (top) and feature importance bar plot (bottom) for the overall test set (including slabs and clusters models). (b, c) SHAP plots and feature (besides EEF) importance bar plots for (b) clusters with GCN < 5 and (c) clusters with GCN > 5.

To understand different factors influencing LEF predictions, we employed SHAP (SHapley Additive exPlanations) analysis.^45^ The EEF consistently emerged as the most significant feature, playing a dominant role in LEF predictions (Figure 6a). However, the importance of other features varied depending on models. To better isolate the influence of geometric factors, we conducted an analysis excluding EEF. For smaller clusters (size <3 nm) at low GCN values (GCN < 5), cluster size had a stronger impact on LEF predictions than GCN (Figure 6b). While for the small clusters (size <3 nm) with higher GCN values (GCN > 5), GCN’s influence became more comparable to that of cluster size (Figure 6c). Overall, the EEF consistently governs LEF predictions, with geometric factors like size and GCN shaping secondary refinements. Understanding these relationships can help guide catalyst design in field-enhanced catalysis so that both GCN and size can be adjusted to tune the LEF.

Our ML model significantly improves computational efficiency in predicting LEFs. For instance, calculating the LEFs from DFT-only calculations using DFT-calculated VSE method required approximately 7674.77 GPU hours, including CO adsorption and vibrational frequency calculations under various EEFs. The PD method required an additional 1973.66 GPU hours, including potential energy calculations over optimized catalyst models under various EEFs. However, our ML model for LEF prediction only requires 0.025 GPU hours, including 0.08 GPU hours for SHAP analysis, 0.017 GPU hours for model training, and hyperparameter optimization. For all 790 data points, this saves approximately 5 orders of magnitude in computational time compared to DFT-only calculations. All computations were performed on the Perlmutter supercomputing cluster at NERSC, utilizing NVIDIA A100 GPUs.

#### 2.3.2 DFT-Based ML Model for Predicting Field-Dependent Adsorption

Field-dependent adsorption energies are essential for understanding the thermodynamics of field-enhanced catalysis. The adsorption energy changes raised by fields can potentially enhance the reaction rates of desired reactions, boosting energy efficiency by orders of magnitude compared to conventional thermal heating.^36^ However, obtaining field-dependent adsorption energies using DFT calculations is computationally expensive.^7^ To address this challenge, we developed two ML frameworks to predict field-dependent adsorption efficiently and accurately, including the DFT-based ML model and physics principles enhanced ML model (Figure 4). Here we used field-dependent CO adsorption as a case study. CO adsorption is critical in many key processes, including CO_2_ electroreduction, Fischer–Tropsch synthesis, methane steam reforming, and water gas shift reactions.^46^ A better understanding of field-dependent CO adsorption could greatly contribute to advancing renewable energy technologies and sustainability efforts.^1,47−50^

For the DFT-based ML model, our data set consists of 717 data points of field-dependent CO adsorption, each containing the external electric field (EEF, V/Å), generalized coordination number (GCN), cluster size (nm), and zero-field adsorption energy (*E*_ad_ (EEF = 0 V/Å), eV) as input features. The EEF ranges from −0.5 to 0.5 V/Å in increments of 0.1 V/Å, covering a broad set of conditions.

We considered three main training data selection scenarios: (1) EEF = ±0.3 V/Å; (2) EEF = ±0.3, ±0.5 V/Å; and (3) a standard random data split of the entire data set (80% training, 10% validation, 10% testing). By choosing suitable data-splitting strategies and ML models, we aimed to achieve accurate and efficient predictions of the adsorption energies under various EEFs. For the first two targeted scenarios, we grouped all data points at the chosen field conditions and allocated 60% for training, 20% for validation, and the remaining 20%—combined with data from other EEF conditions—for testing. This ensures that the test set provides a broad range of conditions, reducing the number of DFT calculations under the EEFs while maintaining predictive accuracy. Figure S17 illustrates these data-splitting methods.

**Figure 7:**
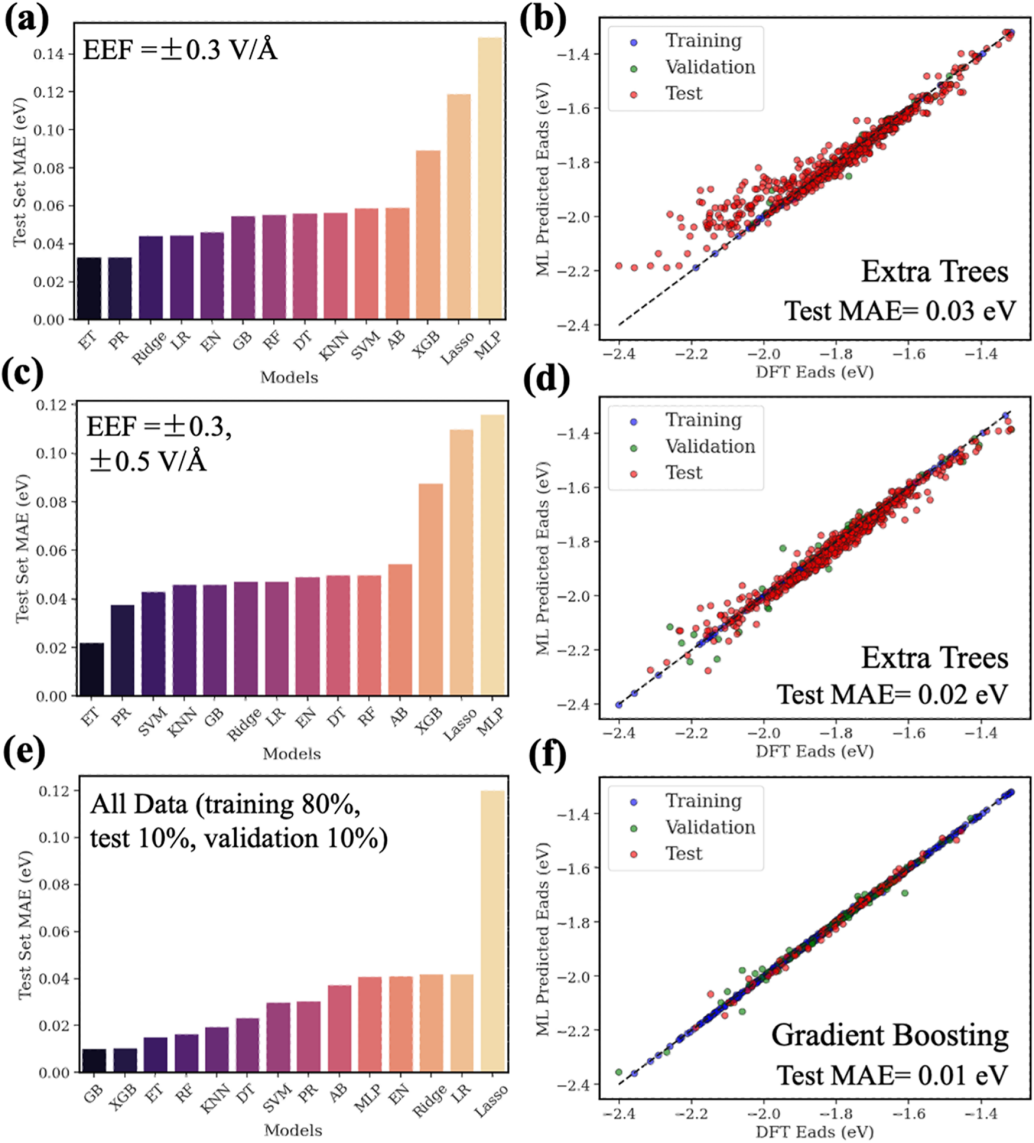
DFT-based ML models performances for different selection methods of training data. (a) Test MAE comparison for different ML models in predicting adsorption energies in the presence of EEFs using training data of EEF = ±0.3 V/Å, (c) training data of EEF = ±0.3, ±0.5 V/Å, and (e) training data of all considered EEFs. (b, d) Comparison between DFT-calculated energetics and the Extra Trees model’s predictions and (f) the XGB model’s predictions. The models in this ML study include gradient boosting (GB), XGBoost (XGB), extra trees regressor (ET), random forest (RF), polynomial regression (PR), support vector regression (SVM), multilayer perceptron (MLP), k-nearest neighbors (KNN), decision tree (DT), AdaBoost (AB), linear regression (LR), and ridge regression (Ridge).

Under an EEF of ±0.3 V/Å, we split 717 data points into 87 training samples, 29 validation samples, and 601 test samples. Under these conditions, the Extra Trees model delivered the best accuracy, achieving a test data MAE of approximately 0.0330 eV (Figure 7a,b). Expanding the range to include ±0.5 V/Å (i.e., EEF= ±0.3, ±0.5 V/Å) increased the training set to 173 and the validation set to 58, while it reduced the test set to 486. This broader training range further improved the Extra Trees model’s performance, yielding a test data MAE of about 0.0222 eV (Figure 7c,d). Finally, when we applied a standard random split of the full data set (573 data for training, 72 data for validation, 72 data for test), a Gradient Boosting (GB) model offered the best overall accuracy with a test data MAE of 0.0103 eV (Figure 7e,f). Although this approach achieved higher accuracy, it required substantially more training data and, consequently, more DFT computational cost at various EEFs.

In terms of computational cost, conducting 717 field-dependent adsorption energetics calculations would require 5791.55 GPU hours. Random splitting of 80% of full data set (717 field-dependent adsorption energetics data points) entailed about 4633.56 GPU hours. Meanwhile, the subsets—(1) EEF = ±0.3 V/Å; (2) EEF = ±0.3, ±0.5 V/Å—reduced data preparation time to 947.77 and 1579.53 GPU hours, respectively. Although these splitting strategies yield slightly larger MAEs than the random-split scenario, they substantially reduce the cost of additional DFT sampling. Once the data set was prepared, the DFT-based ML model training and hyperparameter tuning for Extra Trees required 0.005 and 0.006 GPU hours under EEF = ±0.3 V/Å and EEF = ±0.3, ±0.5 V/Å, respectively. In all cases, these training times constitute only a minor fraction of the total computational cost. Thus, strategically selected EEF conditions achieve near-optimal accuracy while significantly reducing the computational burden for field-dependent adsorption predictions. Tables S7 and S8 and Figures S17 and S18 provide a summary of data sizes, splitting strategies, computational time, model performance, and learning curve. Hyperparameter optimization of DFT-based ML models is shown in Tables S9–S11.

#### 2.3.3 Physics Principles Enhanced ML Model for Predicting Field-Dependent Adsorption

The prediction accuracy of the DFT-based ML model is constrained by the amount of training data. To address this limitation, we integrated the physics principles (i.e., the first-order Taylor expansion for field-dependent adsorption energy estimation^7,37,51,52^) as a training input, aiming to enable predictions with higher accuracy at a reduced training data set compared to DFT-based ML model.

Since the polarizability (second-order coefficient of Taylor expansion) of CO has a relatively small contribution to the field-dependent adsorption compared to its dipole moment,^26,53^ we focused on the first-order Taylor expansion for field-dependent CO adsorption,
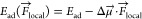
3where *E*_ad_ represents the adsorption energy without EEFs and Δ*μ⃗* denotes the effective dipole moment (approximately −0.10 e·Å, reflecting their charge separation of the species in gas phase, the estimation is shown in Figure S19a).

**Figure 8:**
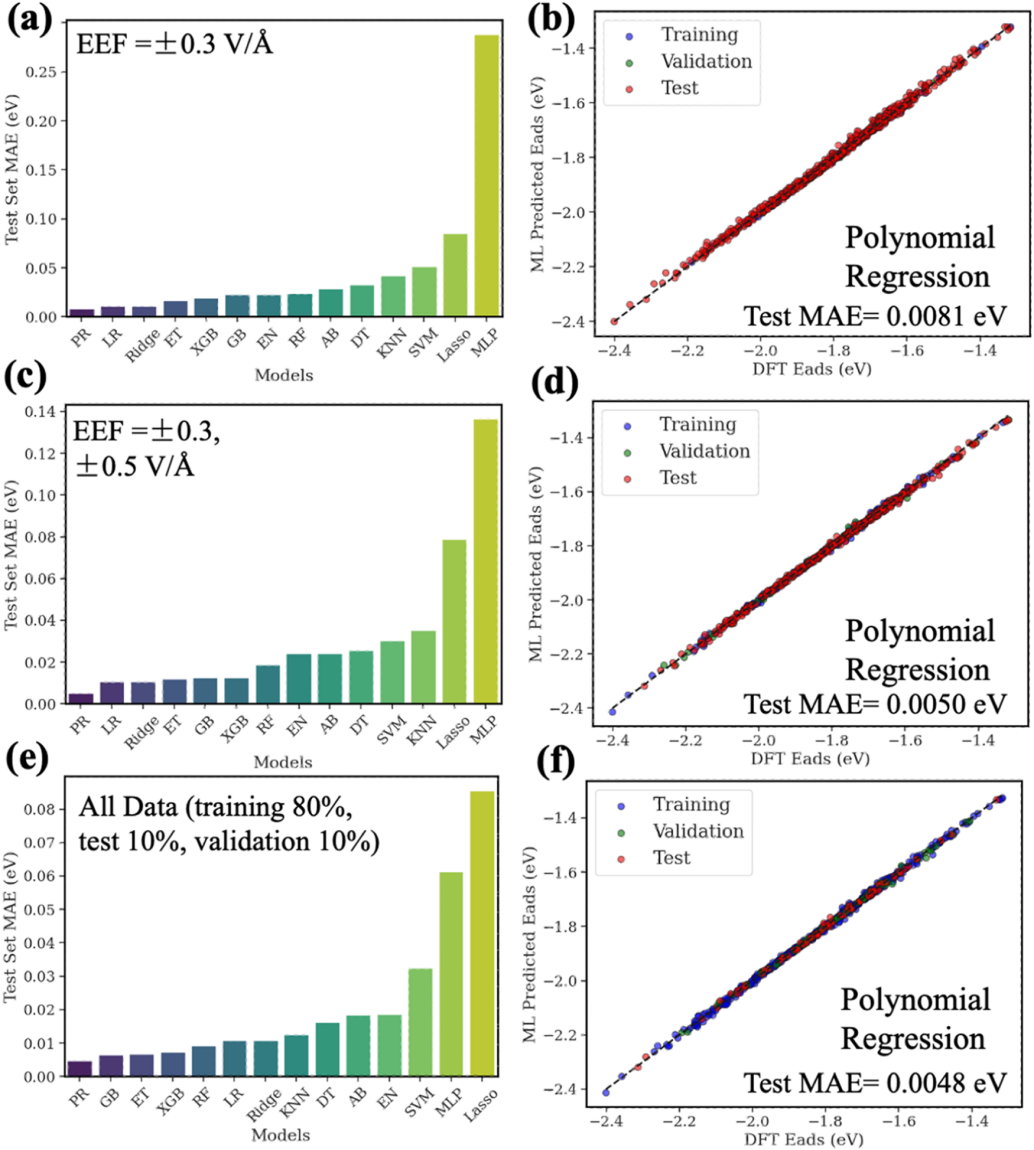
Physics principles enhanced ML models performance for different selection methods of training data. (a) Test MAE comparison for different ML models in predicting adsorption energies in the presence of EEFs using training data of EEF = ±0.3 V/Å, (c) training data of EEF = ±0.3, ±0.5 V/Å, and (e) training 80% data of all considered EEFs. (b, d, f) Comparison between DFT-calculated energetics and the polynomial regression model’s predictions. The ML models include gradient boosting (GB), XGBoost (XGB), extra trees regressor (ET), random forest (RF), polynomial regression (PR), support vector regression (SVM), multilayer perceptron (MLP), k-nearest neighbors (KNN), decision tree (DT), AdaBoost (AB), linear regression (LR), and ridge regression (Ridge).

Building upon the physics principles, we further integrated physics principles (Taylor expansion, Figure S19b) as a training input into the ML model to predict the CO adsorption energies under various EEFs. In physics principles enhanced ML model evaluations, polynomial regression (PR) models achieved exceptional performance with all data set splitting scenarios. Applying the same splitting approaches used for the DFT-based ML (Figure 8a–f), the physics principles enhanced ML method consistently performs better than that of DFT-based ML, delivering lower MAEs. For instance, under the EEF = ±0.3 V/Å scenario (Figure 8a,b), the PR model achieved a test MAE of about 0.0056 eV. Expanding to include EEF = ±0.5 V/Å (Figure 8c,d) further improved the test data set MAE to approximately 0.0044 eV. With a standard random split of 80% of the entire data set as the training data set (Figure 8e,f), the physics principles enhanced ML approach maintained excellent accuracy (test data set MAE = ∼0.0052 eV). Tables S7 and S8 and Figure S17 provide a summary of data sizes, splitting strategies, computational time, and model performance. Hyperparameter optimization of physics principles enhanced ML models is shown in Tables S12–S14.

The learning curves (Figure S18) show that integrating the physics principles to ML model achieves lower MAEs with significantly smaller training data set compared to the DFT-based ML approach. Under EEF =  ±0.3 V/Å, using only ∼22% of the data (∼33 points, 315.92 GPU hours DFT data preparation time) already reduces the test MAE to ∼0.04 eV. Extending to EEF =  ±0.3, ±0.5 V/Å, a similarly sized subset (∼65 points, 526.54 GPU hours DFT data preparation time) delivers an ∼0.0080 eV test MAE—surpassing even the best performance achieved by training on random splitting of 80% of full data set using the DFT-based ML model. Meanwhile, a random-split approach (∼22% of the data, ∼162 points) yields a 0.0056 eV test MAE, but requires 1158.39 GPU hours DFT data preparation time, underscoring a higher computational cost. Overall, these results confirm that the physics principles enhanced ML method can match or exceed the accuracy of the DFT-based model while substantially reducing the total computational expense for DFT data preparation.

Our analysis (Table S15) reveals that the *R*^2^ value between *E*_ads_ (Taylor Expansion Equation Predicted) and DFT-calculated *E*_ads_(*F*) is 0.8131. However, this value significantly improves to 0.9965 for *E*_ads_ (enhanced ML). The *R*^2^ values suggest that while adsorption energy estimation using Taylor Expansion aligns with DFT-calculated adsorption energy, it lacks the accuracy needed for precise adsorption energy prediction when used as a standalone feature. To further understand the role of each input feature in predicting field-dependent CO adsorption with the enhanced ML model, we conducted a Pearson correlation analysis (Figure S20). In this analysis, the Taylor expansion-estimated field-dependent adsorption energy emerged as the most influential factor (*r* = 0.97), underscoring the success of embedding physical principles into the model. In comparison, the zero-field CO adsorption energy (*r* = 0.72) and EEF strength (*r* = 0.63) exhibited moderate correlations, while geometric factors such as GCN (*r* = 0.37) and cluster size (*r* = 0.19) showed weaker, yet non-negligible, correlations.

#### 2.3.4 Transferability of the ML Models

##### 2.3.4.1 Predicting LEFs Using Other Metals

**Figure 9:**
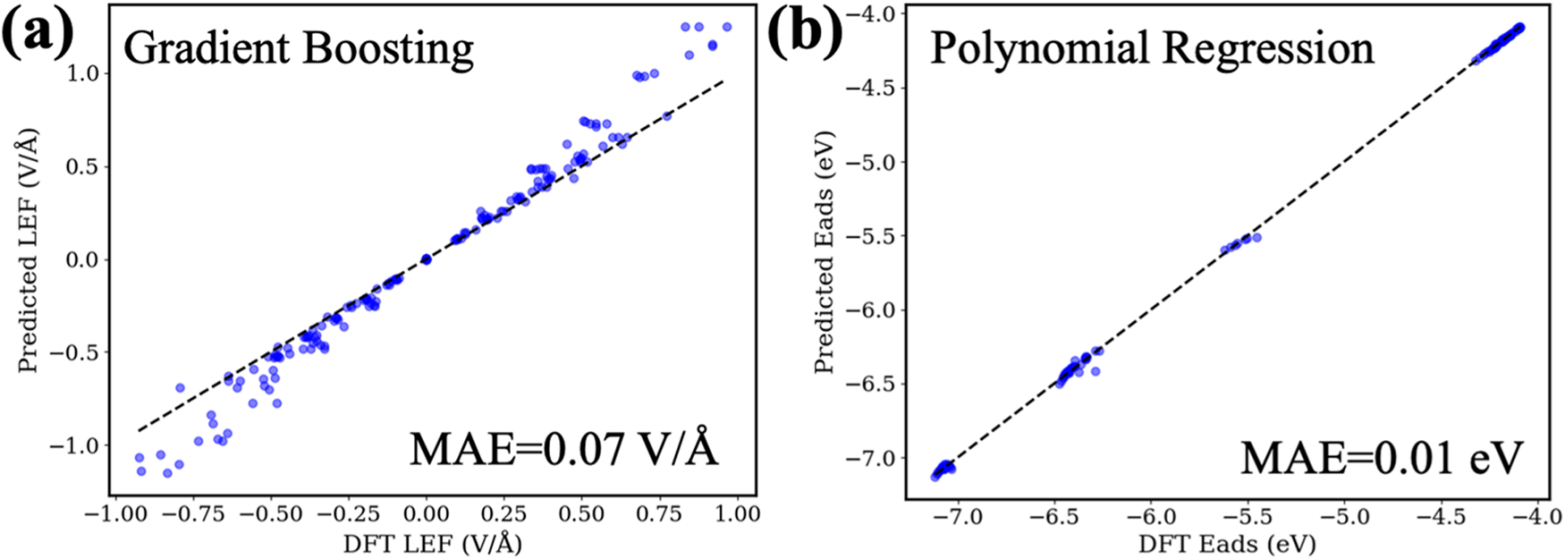
(a) Performance of Ni-trained ML model for predicting the LEFs over Ir NPs/slabs which are not in the Ni training data set. (b) The performance of using the CO-trained physics principles enhanced ML model to predict field-dependent CH adsorption on Ni clusters/slabs. The CO-trained physics principles enhanced ML model is polynomial regression model (with training data set of EEF = ±0.3 V/Å).

To validate the transferability of our ML model for predicting the LEFs over NPs/slabs, we apply Ni-trained ML model to predict the LEFs over the Iridium (Ir) NPs/slabs—a completely different metal system. The model achieved excellent performance with a MAE of 0.0737 V/Å across 181 new Ir data points, which have never been seen in our Ni data set (Figure 9a). This demonstrates our ML framework’s ability to predict the LEFs of other metal systems at a given catalyst characteristics (GCN, Size) and an EEF without requiring additional DFT calculations. Table S16 and Figure S21 illustrate the performance of linear regressions for correcting PD-mapped LEFs by using DFT-based VSE methods.

##### 2.3.4.2 Predicting Field-Dependent Adsorption Using Other Adsorbates

To validate our physics principles enhanced ML model’s transferability, we predict the field-dependent CH adsorption energies on the Ni top sites, an entirely different adsorbate system from our CO-Ni system. We applied the CO-trained physics principles enhanced ML model to predict the field-dependent CH adsorption energetics (unseen from ML data set). The MAE is 0.01 eV (Figure 9b). This indicates that our physics principles enhanced ML approach has the transferability to other unseen system to fast and accurately predict the field-dependent adsorption energetics.

## 3 Conclusions

In this study, we have developed DFT-based ML and physics principles enhanced ML models to predict the local electric field distribution and its impact on adsorption properties for advancing field-enhanced catalysis. Our PCA and SHAP analysis reveal that the external electric fields, the generalized coordination number, and the cluster size are the dominant factors governing the local electric field distribution. Due to the charge accumulation at low-coordinated sites over small clusters, those sites have larger local electric fields compared to the extended slabs. Using gradient boosting regression, we can predict the local electric field distribution quickly at a given catalyst geometry under certain external electric fields. We then integrate the physics principles (Taylor expansion) as training inputs into the ML models to predict field-dependent adsorption energy. The physics principles enhanced ML model has higher accuracies at a reduced training data set as compared to the ML model without embedding the physics (DFT-based ML model). Both ML models for predicting LEFs and the physics principles enhanced ML model for predicting the field-dependent adsorption energetics have the transferability to other unseen systems. The physics-enhanced ML algorithm offers a novel approach for rapidly and accurately predicting field-dependent adsorption with a limited training data set, paving the way for designing field-enhanced catalysts. This method holds particular promise for electrified technologies characterized by strong local electric fields, including plasma catalysis, electrostatic catalysis, programmed catalysis, electrocatalysis, and fuel cells. The insights and open-source ML models developed in this work can be extended to tackle more complex challenges, such as olefin polymerization/oxidation, biomass conversion, alkane activation, nitrogen fixation, and CO_2_ fixation under electric field effects.

## 4 Methodology

### 4.1 DFT Calculations and Electric Field Implementation

All density functional theory (DFT) calculations were performed using Vienna Ab initio Simulation Package (VASP).^54^ The Perdew–Burke–Ernzerhof (PBE) functional^55^ was applied for the exchange and correlation energy of electrons. The projector augmented wave (PAW) method^56^ was applied to accurately describe core–valence electron interactions. The kinetic energy cutoff of 400 eV was applied for the plane-wave basis set. The configurations were optimized until the maximum force and energy difference between each self-consistency loop on each atom were less than 0.03 eV/Å and 10^–4^ eV, respectively.^12^ Spin-polarization with the initial guess of magnetic moment of 2 per Ni atom was included in all calculations to accurately describe the magnetic properties of the Ni slabs and nanoparticles. External electric fields (EEFs) were implemented using the Neugebauer and Scheffler method,^38^ which introduces a dipole sheet with opposite charges at the simulation cell boundaries including the slab surfaces and the clusters. EEFs ranging from −0.5 to 0.5 V/Å were applied in increments of 0.1 V/Å, with the field direction perpendicular to the surface plane.^37^

### 4.2 Model Systems

Two types of Ni models were employed in this study: nanoparticle (NP) clusters^57^ and surface slabs. Ni lattice constant is 3.524 Å.^12^ The NP cluster models ranged from 0.76 nm (Ni_38_) to 1.53 nm (Ni_201_) in diameter, corresponding to both experimentally observed morphologies and theoretically predicted low-energy structures.^57,58^ For the NPs, a 40 Å vacuum space was applied along the *x* and y axes to avoid interactions between NP structures.^57,58^ A vacuum space of 13.6 Å was implemented in all directions to prevent interactions between periodic images, and 1 × 1 × 1 k-point sampling was used for all cluster calculations.

Surface models represent^59^ larger NPs (>3 nm). *p*(4 × 4) supercells were constructed for the thermodynamically favorable low-index surfaces ((111), (100)) and *p*(2 × 4) supercells were constructed for stepped surface (211). These slab models consisted of four atomic layers, with the bottom two layers fixed at bulk positions and the top two layers allowed to fully relax. A vacuum spacing of 13.6 Å was maintained perpendicular to the surface, and a 3 × 3 × 1 *k*-point mesh was employed.^60^

### 4.3 Generalized Coordination Number

And the local coordination environment was considered and described by the generalized coordination number (GCN)^61,62^ as shown in eq 4.
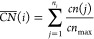
4where *cn*(*j*) represents the coordination number of the *j* nearest neighbor of atom *i* and *cn*_max_ is the maximum coordination number in the bulk material. By taking into account the coordination of both the central atom and its neighbors, the GCN provides a nice representation of the local structure of the active site, capturing how the subtle variations in active site configurations (defects, shape, sizes) will influence local electric field distribution and, subsequently, alter the performance of catalytic reactions where large field exists.

### 4.4 Adsorption Energy Calculations

To investigate the adsorption properties and vibrational frequencies of CO on the cluster and slab models with and without field effects, CO was placed at top adsorption sites, which are the favorable adsorption sites in most of our Ni clusters.^63,64^ The adsorption energy of CO on the catalyst surfaces is calculated using the following equation:
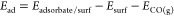
5where *E*_adsorbate/surf_, *E*_surf_, and *E*_CO(g)_ represent the total energies of the CO-adsorbed surface/NP, the clean surface/NP, and the gas-phase CO molecule, respectively. The finite difference method with a displacement of ±0.01 Å was employed to obtain the vibrational frequencies of adsorbed CO with and without external electric fields. This will allow us to calculate the vibrational stark effects to quantitatively determine the local electric fields.

## Data Availability

The structures and Python scripts can be accessed from our group website: https://github.com/FanglinGroup.

## Supporting Information Available

Computational setup, machine learning models, and local electric fields (PDF)
