# Supplementary material for: Integrating Physical Principles with Machine Learning for Predicting Field-Enhanced Catalysis
Source: JACS Au. 2025 Feb 17;5(3):1121–32. doi: 10.1021/jacsau.4c00901 (PMC11938032; doi:10.1021/jacsau.4c00901)
Supplement: Supplementary file 1 — au4c00901_si_001.pdf [file au4c00901_si_001.pdf]

Support Information

Integrating Physical Principles with Machine Learning for Predicting Field-Enhanced Catalysis

Runze Zhao[1], Qiang Li[1], Jiaqi Yang[1], Cheng Zhu[2], Fanglin Che[1]*

[1]*Department of Chemical Engineering, University of Massachusetts Lowell, USA, 01854*

[2]*Engineering Directorate, Lawrence Livermore National Laboratory, 7000 East Ave, Livermore, CA 94550, USA*

Corresponding Information: *fanglin_che@uml.edu

Table of Content

1. Computational Setup

   1.1 Model Systems

   Figures S1-S4. Structural configurations of Ni surfaces and nanoparticles.

   1.2 CO Adsorption

   Figures S5-S10. CO adsorption configurations on Ni surfaces and nanoparticles.

2. Correction of local electric field mapped by potential difference (PD) method using DFT-calculated vibrational stark effect

   Tables S1-S2. Models' performance for the PD and VSE correction for slabs and clusters.

3. Differential charge density analysis

   Figure S11. Top views of differential charge density plots

4. Machine Learning

   4.1. Local Electric Field (LEF) Predictions

   Table S3. GCN and LEF for various Ni slab surfaces and nanoparticles.

   4.2 DFT Database

   Figures S12-S15. Data availability heat maps for Ni surfaces and nanoparticles.

   4.3 ML Input Features

   Table S4. Input Features used for predicting LEFs.

   Table S5. Input Features used for predicting adsorption energy with external electric fields (EEF).

   4.4 Local Electric Field (LEF) Prediction Learning curve and Optimized Hyperparameters

   Figure S16. Learning curves for ML in predicting LEF.

   Table S6. Optimized hyperparameters of ML models for local electric field prediction

   4.5 Data Selections, Splitting, and Learning Curves for Field-Dependent Adsorption Prediction

   Table S7. Data splitting strategies for predicting adsorption energy with EEF

   Table S8. Different training data selection and performance for predicting adsorption energy with EEF.

   Figure S17. The data splitting method for predicting the adsorption energy in the presence of EEF.

   Figure S18. Learning curve for training MAE and test MAE when predicting field-dependent adsorption energies.

4.6 Taylor expansion for Field-Dependent Adsorption

Figure S19. Effective dipole moment of CO and performance of the first order Taylor expansion in predicting field-dependent CO adsorption energies.

4.7 Field-Dependent Adsorption Prediction and Optimized Hyperparameters

Tables S9-S11. Optimized hyperparameters of DFT-based ML models for field-dependent CO adsorption energetics prediction.

Tables S12-S14. Optimized hyperparameters of physics principles enhanced ML models for field-dependent CO adsorption energetics prediction.

Table S15. R^2 of different models in predicting field-dependent adsorption energies.

4.8 Pearson Correlation Analysis for the Physics-Principles-Enhanced ML Model

Figure S20. Pearson Correlation between input features for physics principles enhanced ML model for the scenarios of EEF = ±0.3, ±0.5 V/Å.

4.9 Transferability of the ML Models

Table S16. Performance of linear regression for correcting PD-mapped LEFs using VSE for Ir system.

Figure S21. (a)The LEF of the top site of Ir(100) using VSE method (b) PD-mapped LEF of the top of Ir(100), via VSE corrections.

1. Computational Setup

1.1 Model Systems

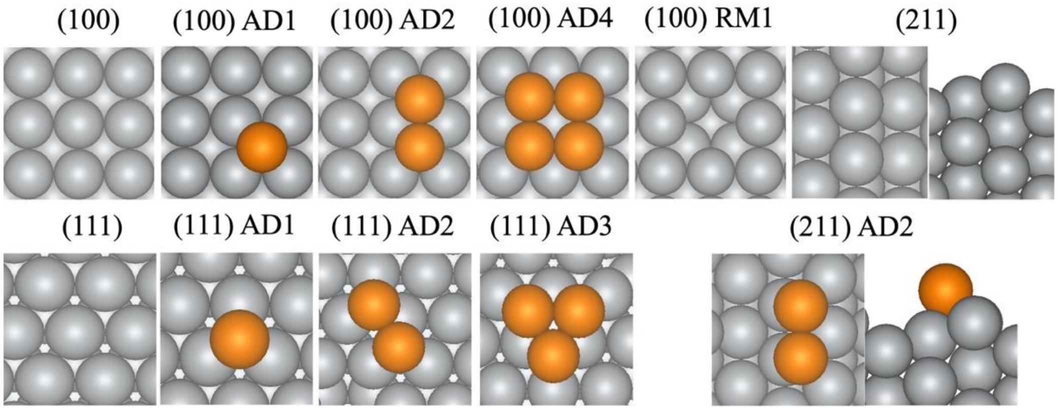

Figure S1. Top views of various slab surface models used in this study. Top row (left to right): (100) clean surface, (100) with one adatom (AD1), (100) with two adatoms (AD2), (100) with four adatoms (AD4), (100) with one atom removed (RM1), and (211) stepped surface. Bottom row (left to right): (111) clean surface, (111) with one adatom (AD1), (111) with two adatoms (AD2), (111) with three adatoms (AD3), and (211) with two adatoms (AD2). Gray spheres represent surface atoms, while orange spheres indicate adatoms.

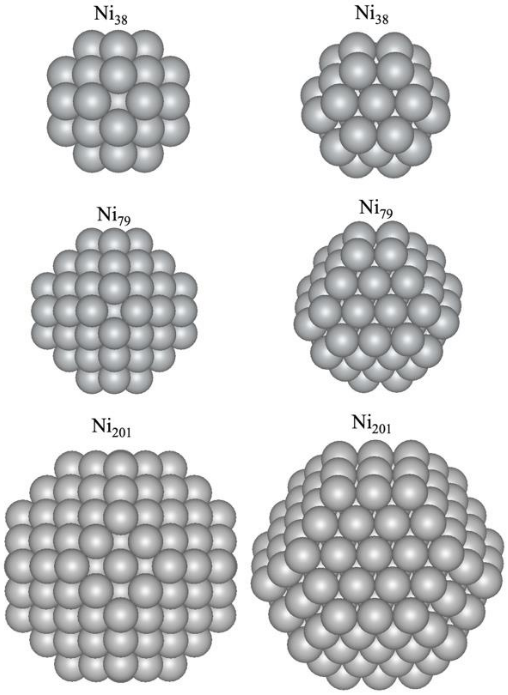

Figure S2. Top and side views of various Ni nanoparticles. Each row shows a different size nanoparticle: Ni_{38} (0.76 nm, top), Ni_{79} (1.08 nm, middle), and Ni_{201} (1.53 nm, bottom).

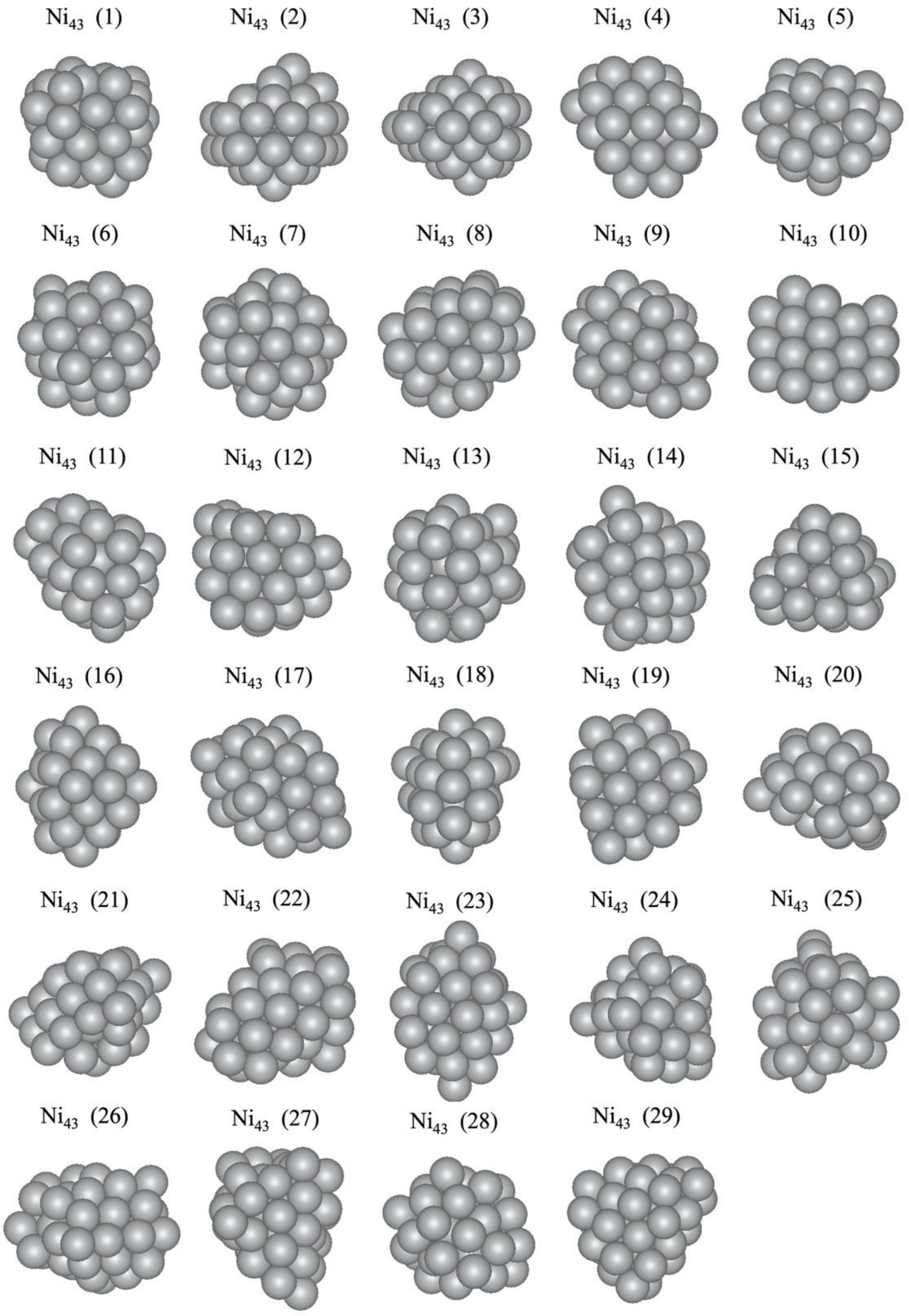

Figure S3. 29 Configurations of Ni_{43} (0.87 nm to 1.09 nm) nanoparticles.

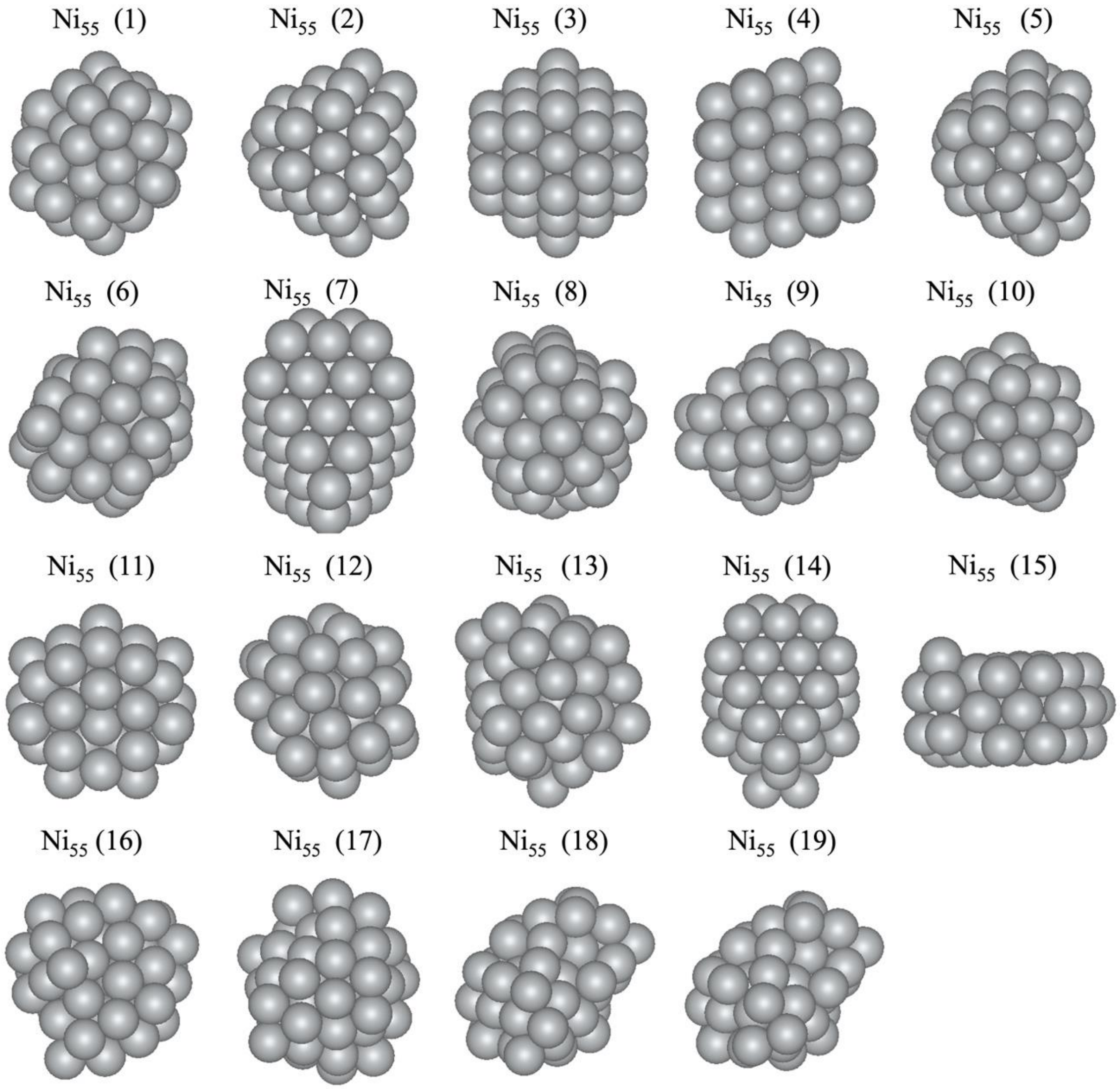

Figure S4. 20 Configurations of Ni_{55} (0.93 nm to 1.19 nm) nanoparticles.

1.2 CO Adsorption

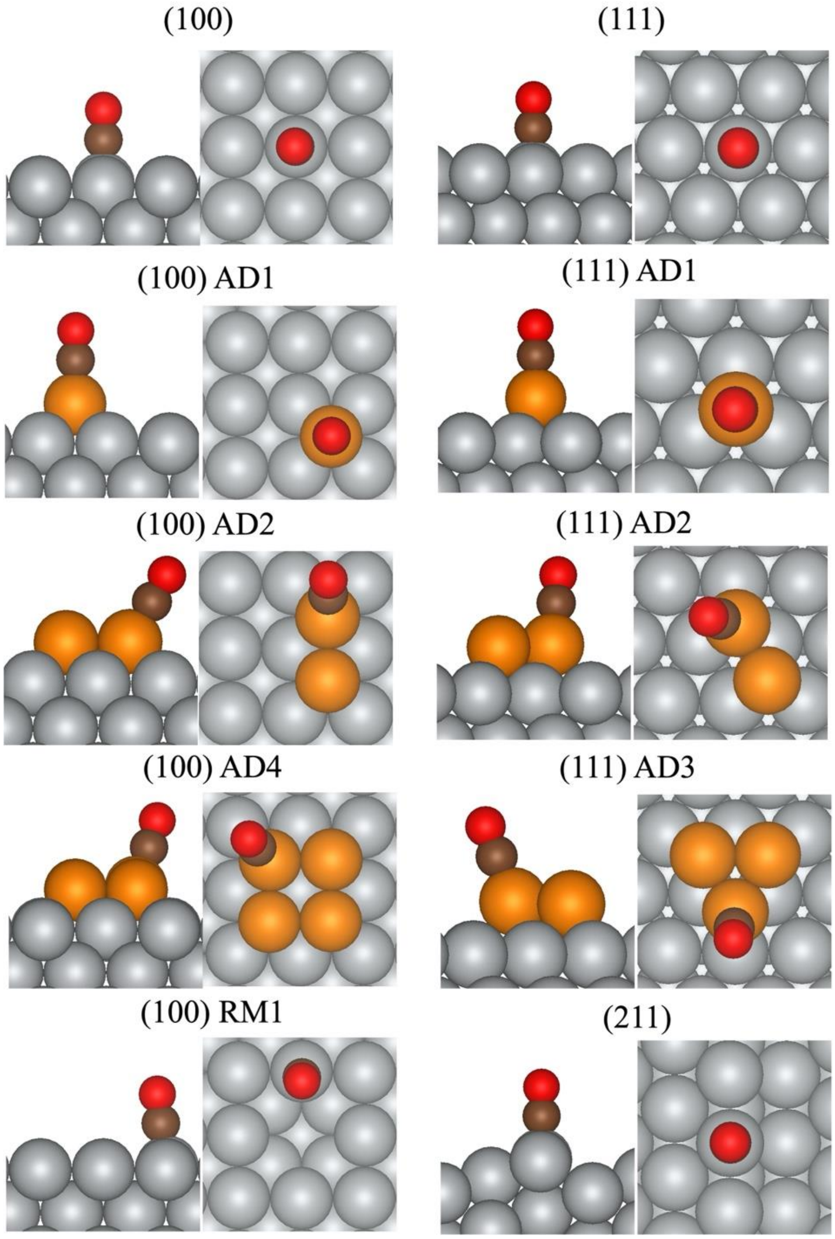

Figure S5. Side and top views of CO adsorption at top sites of slab models. The surface atoms, adatoms, carbon, and oxygen atoms are gray, orange, brown, and red, respectively.

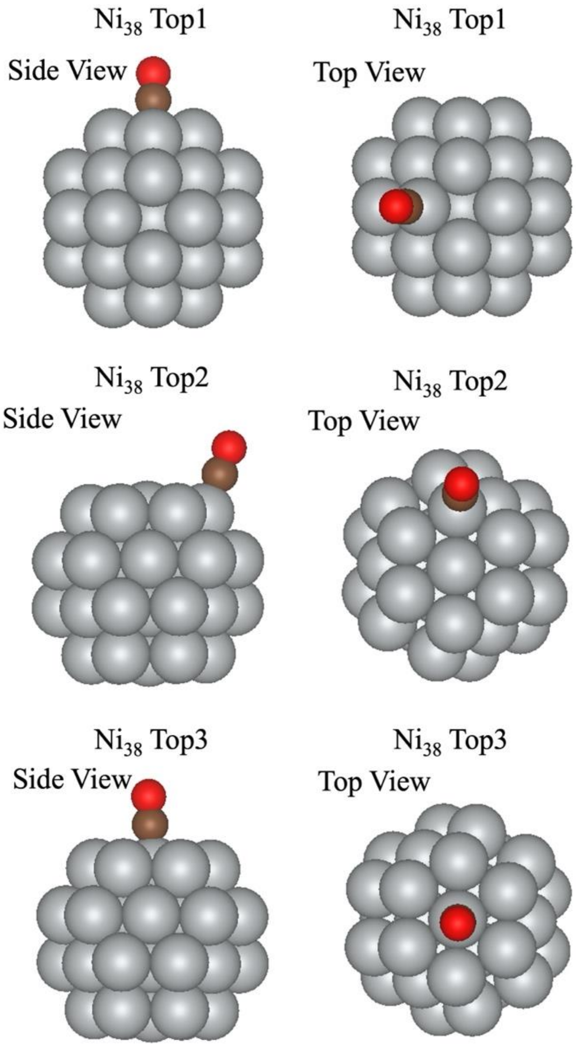

Figure S6. Side and top views of CO adsorption configurations on three top sites (Top1, Top2, Top3) of Ni_{38} (0.76 nm).

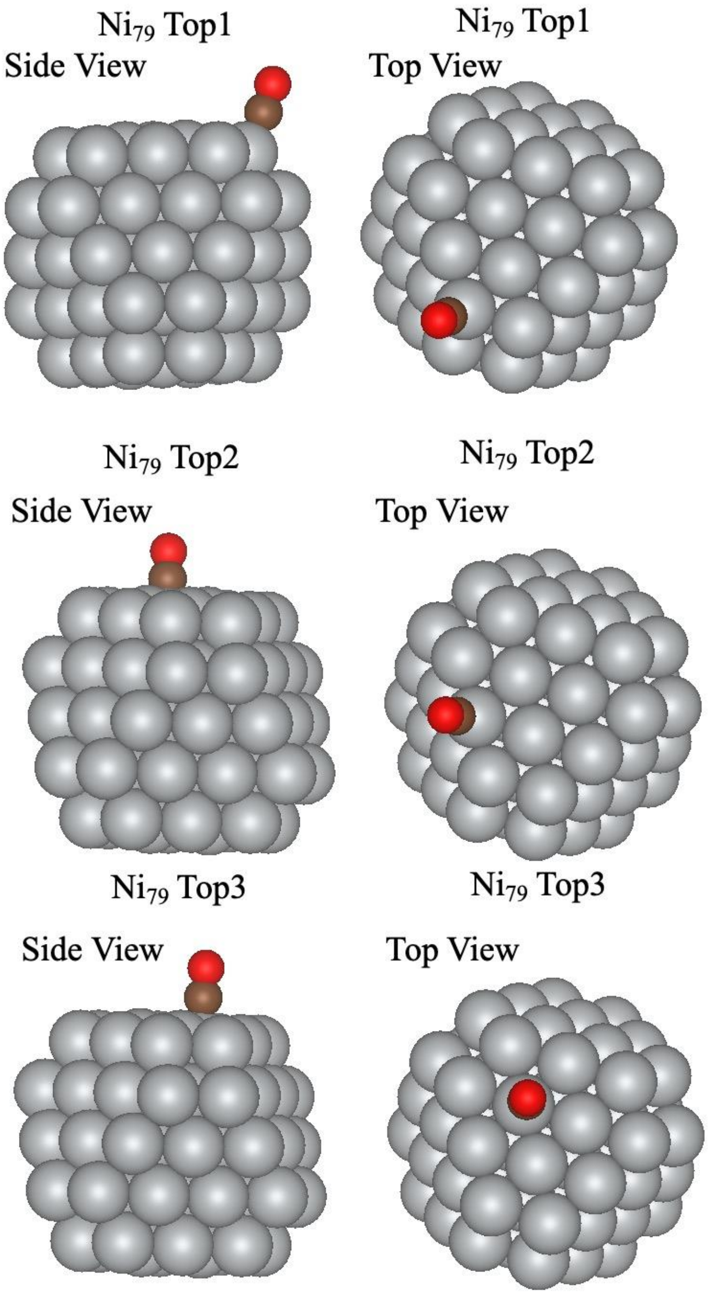

Figure S7. Side and top views of CO adsorption configurations on four top sites (Top1, Top2, Top3) of Ni_{79} (1.08 nm).

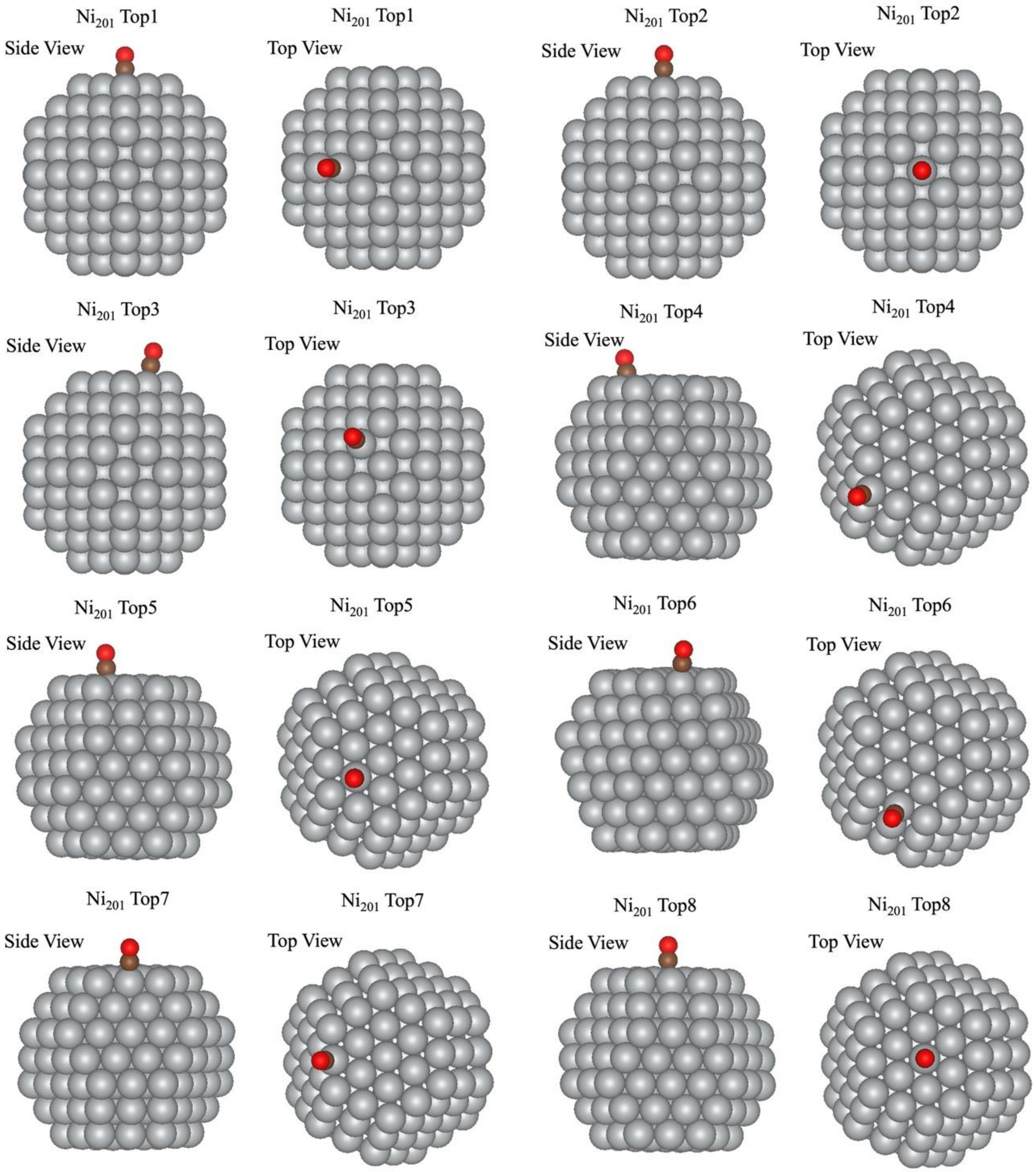

Figure S8. Side and top views of CO adsorption configurations on eight top sites (Top1 to Top8) of Ni_{201} (1.53 nm).

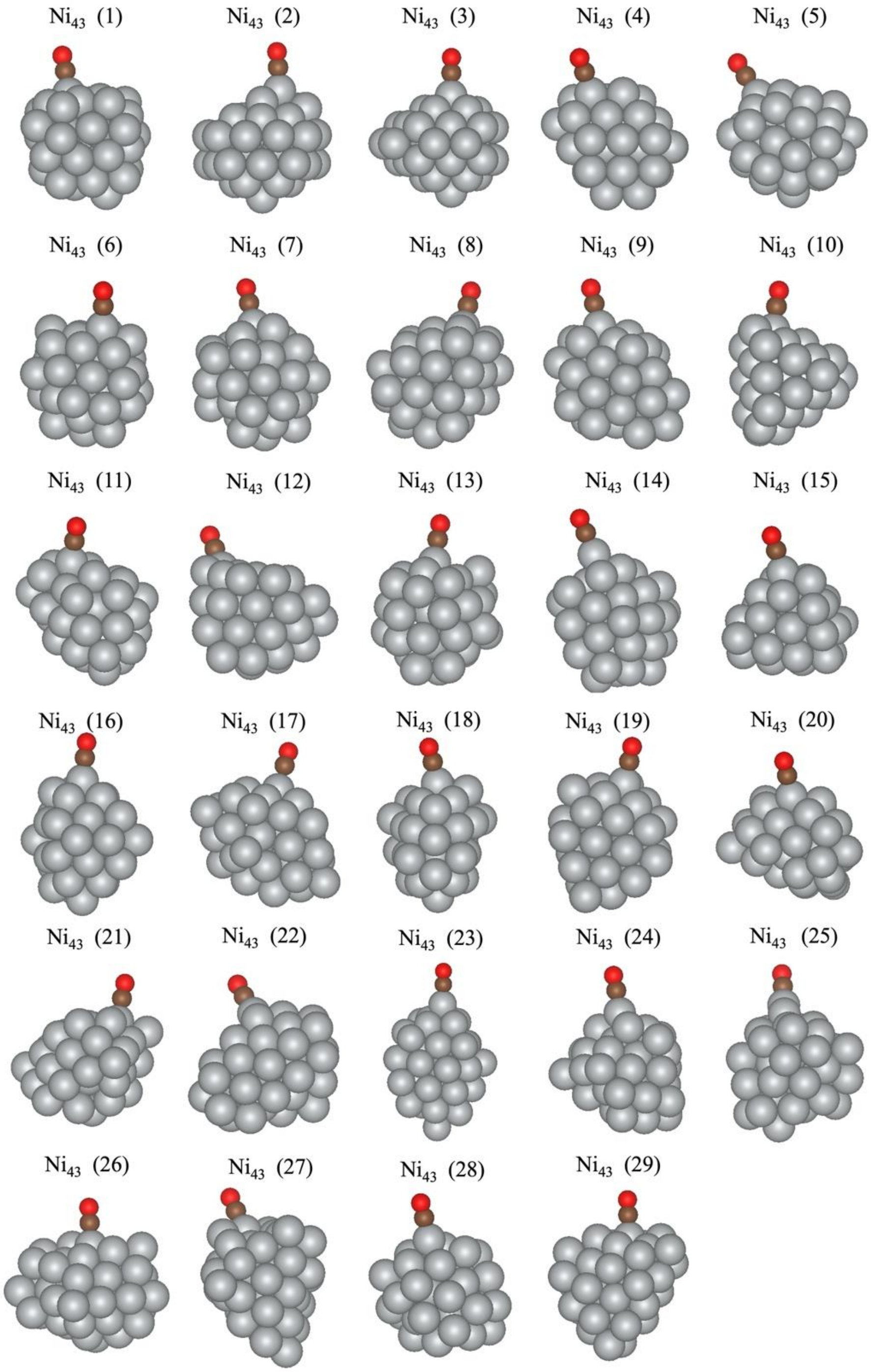

Figure S9. 29 Configurations of CO adsorption over Ni_{43} (0.87 nm to 1.09 nm) nanoparticles.

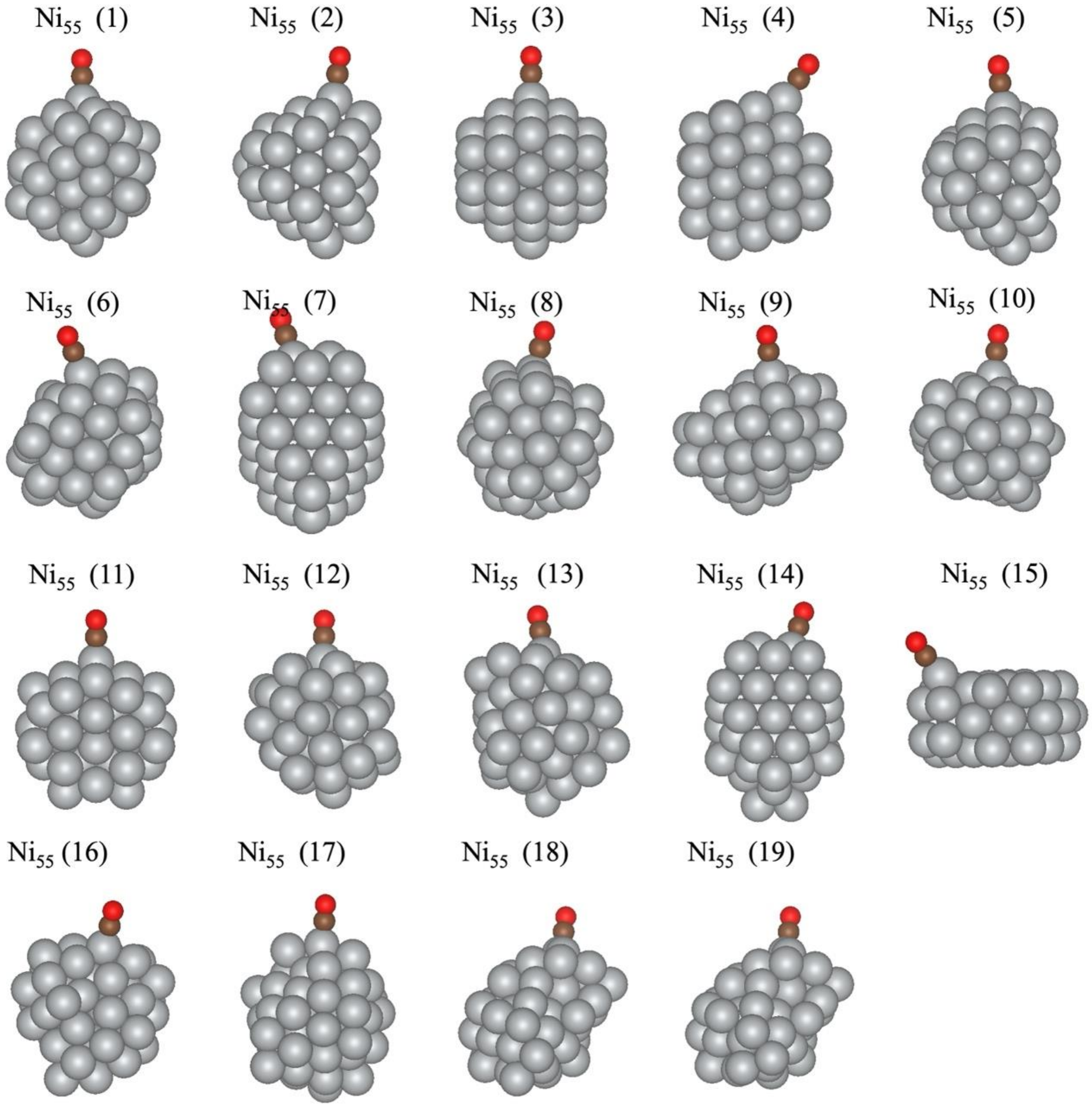

Figure S10. 19 Configurations of CO adsorption over Ni_{55} (0.93 nm to 1.19 nm) nanoparticles.

2. Correction of local electric field (LEF) mapped by potential difference (PD) method using DFT-calculated vibrational stark effect (VSE)

Several studies have established that DFT calculations can reliably predict vibrational Stark effects, often matching experimental observations closely enough to serve as a benchmark for mapping local electric fields (LEFs). For example, Brewer and Franzen calculated the vibrational Stark tuning rates of various nitriles and carbonyls using DFT and found quantitative agreement with experimentally measured local fields, demonstrating the high accuracy achievable by this method.[1] Additionally, Wright et al. combined theoretical and experimental approaches to investigate the vibrational Stark effect in molecules forming monolayers on electrodes. Their findings indicated that applying a uniform electric field in DFT simulations cannot always capture all vibrational modes simultaneously, thereby underscoring the necessity of incorporating local environmental factors—such as ionic migration—to enhance realism.[2] Furthermore, Garrett et al. developed a real-space pseudopotential technique for computing the vibrational Stark effect, showing that careful consideration of anharmonic contributions and applied fields enables ab initio simulations to reproduce VSE phenomena with high fidelity.[3] Taken together, these studies confirm that DFT-based VSE calculations can closely align with experimental results, provided that the measurements of LEFs under various environmental factors can be sufficiently captured within the computational framework.[3] Below, we present the linear regression (LR) and polynomial regression (PR) corrections with various polynomial degrees relating between the PD method to DFT-calculated VSE in our study.

Table S1. Performance of different models for correcting PD-mapped LEFs using VSE on Slab Surfaces

	Equations	MAE (V/Å)	RSME (V/Å)	R^2
LR	$VSE = -0.0266 + 1.1605 \times PD$	0.0541	0.0729	0.9651
PR (2 degree)	$VSE = -0.0054 + 1.1616 \times PD - 0.1943 \times PD^2$	0.0505	0.0701	0.9677
PR (3 degree)	$VSE = -0.0053 + 1.2096 \times PD - 0.1946 \times PD^2 - 0.2320 * PD^3$	0.0505	0.0696	0.9682

Note: Here, MAE, RSME and R^2 are based on the difference between model predictions and LEFs calculated by DFT-based VSE for all data (790 data points).

Table S2. Performance of different models for correcting PD-mapped LEF using VSE on Nanoparticles

	Equations	MAE(V/Å)	RSME(V/Å)	R^2
LR	$VSE = -0.0562 + 1.3272 \times PD$	0.0981	0.1282	0.9744
PR (2 degree)	$VSE = 0.0010 + 1.3310 \times PD - 0.1608 \times PD^2$	0.0856	0.1166	0.9788
PR (3 degree)	$VSE = 0.0010 + 1.3456 \times PD - 0.1605 \times PD^2 - 0.0218 \times PD^3$	0.0855	0.1165	0.9789

Note: Here, MAE, RSME and R^2 are based on the difference between model predictions and LEFs calculated by DFT-based VSE for all data (790 data points).

3. Differential Charge Density Analysis

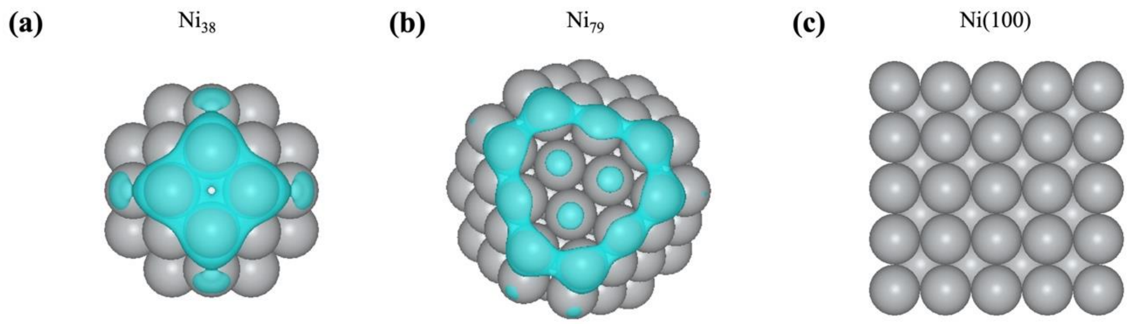

Figure S11. Top views of differential charge density plots (a), (b), and (c) correspond to Ni_{38}, N_{i79}, and Ni(100) when EEF is 0.5 V/Å, respectively. The iso-surfaces are visualized at 0.0005 e/Bohr^3.

4. Machine Learning

4.1. Local Electric Field Predictions

Tables S3 presents the local electric fields (LEF) from VSE, PD and VSE-corrected PD method for various catalyst models when the external electric field (EEF) is 0.5 V/Å.

Table S3. Generalized Coordination Numbers (GCN) and LEF for various Ni surfaces and NPs under EEF of 0.5 V/Å.

Models	GCN	EF (V/Å)	NP Size(nm)	Type	Models	GCN	EF (V/Å)	NP Size(nm)	Type
Ni_{38}(1)	4	1.14	0.76	NP	Ni_{43}(24)	3.17	1.44	1.01	NP
Ni_{38}(2)	4	1.06	0.76	NP	Ni_{43}(25)	1.58	1.21	0.99	NP
Ni_{38}(3)	6	1.15	0.76	NP	Ni_{43}(26)	4.33	1.27	1.01	NP
Ni_{79}(1)	4.08	1.05	1.08	NP	Ni_{43}(27)	3.17	1.08	0.99	NP
Ni_{79}(2)	5	1.02	1.08	NP	Ni_{43}(28)	3	1.29	0.9	NP
Ni_{79}(3)	6.67	1.1	1.08	NP	Ni_{43}(29)	4.5	1.23	1.03	NP
Ni_{201}(1)	4.25	1.24	1.53	NP	Ni_{55}(1)	4.25	1.45	0.96	NP
Ni_{201}(2)	6.33	1.32	1.53	NP	Ni_{55}(2)	3.25	1.47	1	NP
Ni_{201}(3)	5.17	1.32	1.53	NP	Ni_{55}(3)	4	1.5	0.97	NP
Ni_{201}(4)	4.25	1.06	1.53	NP	Ni_{55}(4)	2.5	1.25	1.04	NP
Ni_{201}(5)	6.92	1.13	1.53	NP	Ni_{55}(5)	3.33	1.42	0.97	NP
Ni_{201}(6)	5.17	1.11	1.53	NP	Ni_{55}(6)	4.08	1.23	1.04	NP
Ni_{201}(7)	5	1.05	1.53	NP	Ni_{55}(7)	3.92	1.26	1.12	NP
Ni_{201}(8)	7.5	1.1	1.53	NP	Ni_{55}(8)	4.5	1.33	1.01	NP
Ni_{43}(1)	4.42	1.16	0.88	NP	Ni_{55}(9)	4	1.31	1.01	NP
Ni_{43}(2)	3.08	1.41	0.93	NP	Ni_{55}(10)	3.08	1.41	1.05	NP
Ni_{43}(3)	4.25	1.37	1.02	NP	Ni_{55}(11)	2.25	1.35	0.93	NP

**Models**	**GCN**	**EF (V/Å)**	**NP Size(nm)**	**Type**	**Models**	**GCN**	**EF (V/Å)**	**NP Size(nm)**	**Type**
Ni_{43}(4)	3.92	1.18	0.92	NP	Ni_{55}(12)	4.17	1.27	0.93	NP
Ni_{43}(5)	3.83	1.03	0.91	NP	Ni_{55}(13)	3.25	1.35	1.05	NP
Ni_{43}(6)	4.08	1.24	0.88	NP	Ni_{55}(14)	3.92	1.29	1.17	NP
Ni_{43}(7)	3.92	1.31	0.87	NP	Ni_{55}(15)	2.5	0.88	1.03	NP
Ni_{43}(8)	3.92	1.17	0.9	NP	Ni_{55}(16)	2.83	1.26	1.09	NP
Ni_{43}(9)	3	1.4	0.96	NP	Ni_{55}(17)	3.42	1.49	1.05	NP
Ni_{43}(10)	4.58	1.3	0.91	NP	Ni_{55}(18)	2.5	1.21	1.19	NP
Ni_{43}(11)	4.25	1.25	0.92	NP	Ni_{55}(19)	3.08	1.23	1.14	NP
Ni_{43}(12)	2.83	1.06	0.92	NP	Ni(100)	6.67	0.55		Slab
Ni_{43}(13)	4	1.32	0.89	NP	Ni(100)AD1	3	0.77		Slab
Ni_{43}(14)	2.25	1.43	0.98	NP	Ni(100)AD2	3.58	0.65		Slab
Ni_{43}(15)	3.67	1.24	1.01	NP	Ni(100)AD4	4.42	0.62		Slab
Ni_{43}(16)	3.58	1.48	0.9	NP	Ni(100)RM1	5.83	0.54		Slab
Ni_{43}(17)	3.25	1.3	1.04	NP	Ni(111)	7.5	0.54		Slab
Ni_{43}(18)	3	1.34	1.06	NP	Ni(111)AD1	2.5	0.65		Slab
Ni_{43}(19)	3.83	1.31	1.09	NP	Ni(111)AD2	2.92	0.52		Slab
Ni_{43}(20)	3.08	1.23	0.93	NP	Ni(111)AD3	3.5	0.57		Slab
Ni_{43}(21)	4.5	1.21	0.99	NP	Ni(211)	5.5	0.61		Slab
Ni_{43}(22)	2.25	1.07	1.01	NP	Ni(211)AD2	4.67	0.65		Slab
Ni_{43}(23)	2.33	1.6	1.08	NP					

4.2 DFT Database

Our database consists of 790 datapoints derived from DFT calculations using the VSE-corrected PD method, encompassing local electric fields and CO adsorption energies for nickel catalysts. We examined various cluster and slab models under a range of EEFs from -0.5 V/Å to 0.5 V/Å. Each datapoint includes information on surface structure, cluster size, and the GCN of Ni catalysts. **Figures S12 to S15** visualize our DFT database, showing data availability across different nanoparticle structures and EEF conditions.

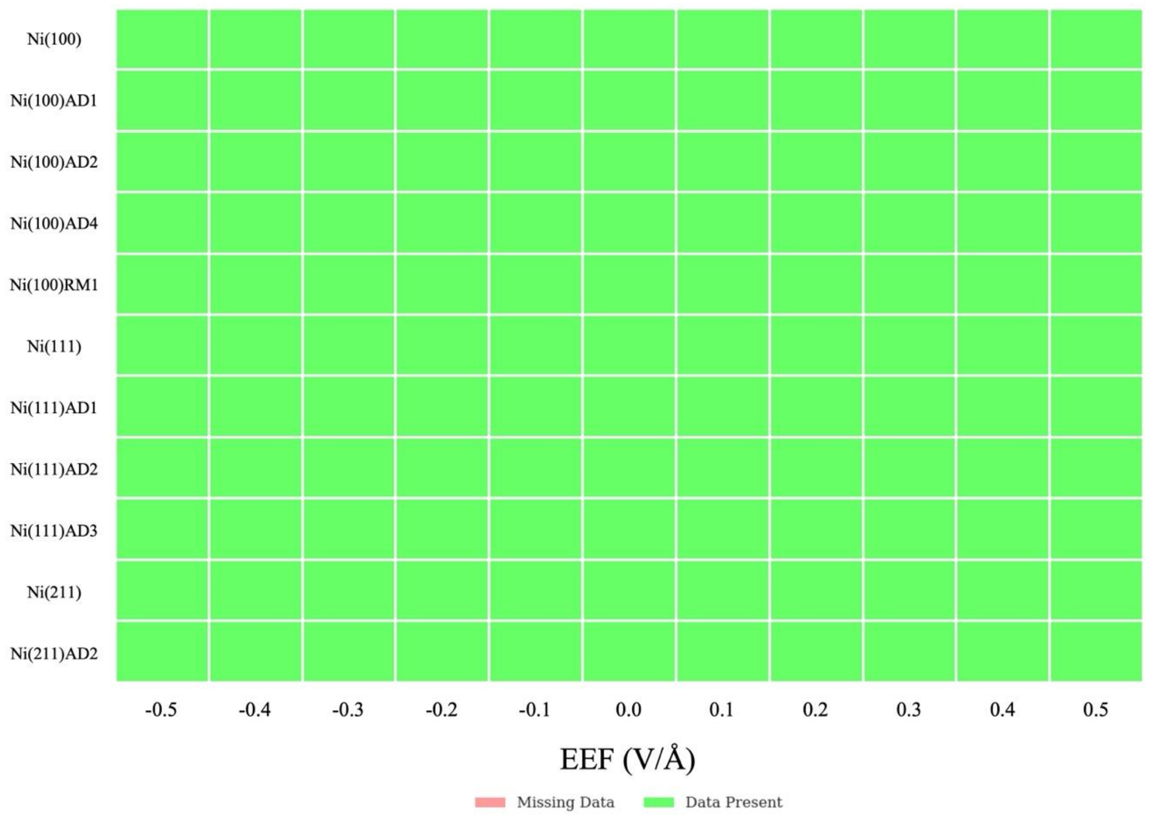

Figure S12. Data availability heatmap for Ni slab models.

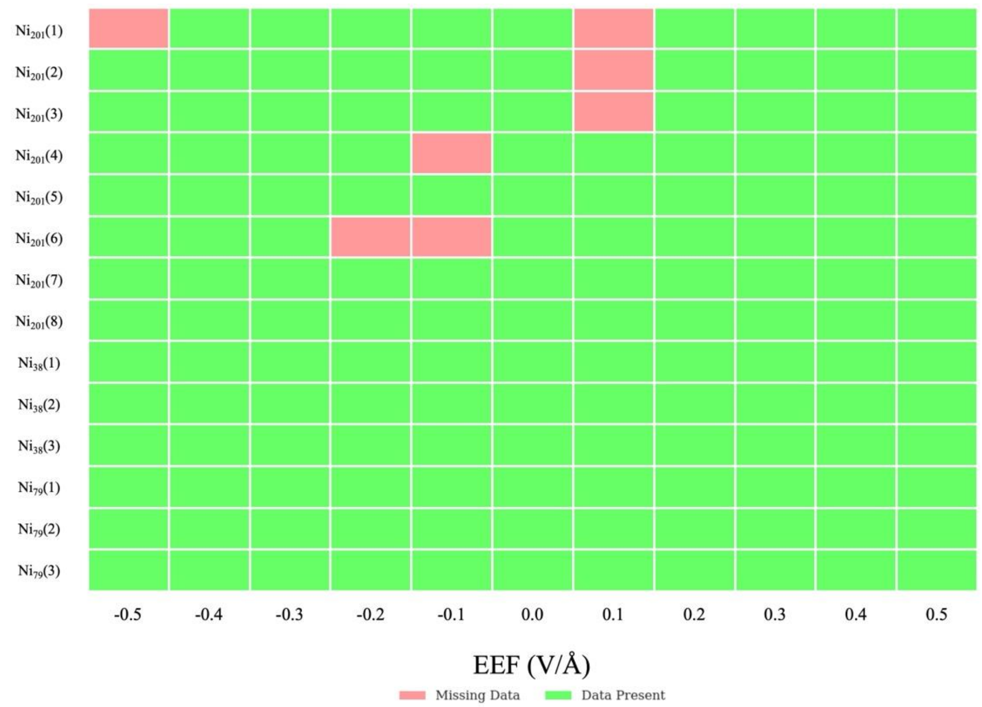

Figure S13. Data availability heatmap for Ni_{201} (1.53 nm), Ni_{38} (0.76 nm) and Ni_{79} (1.08 nm).

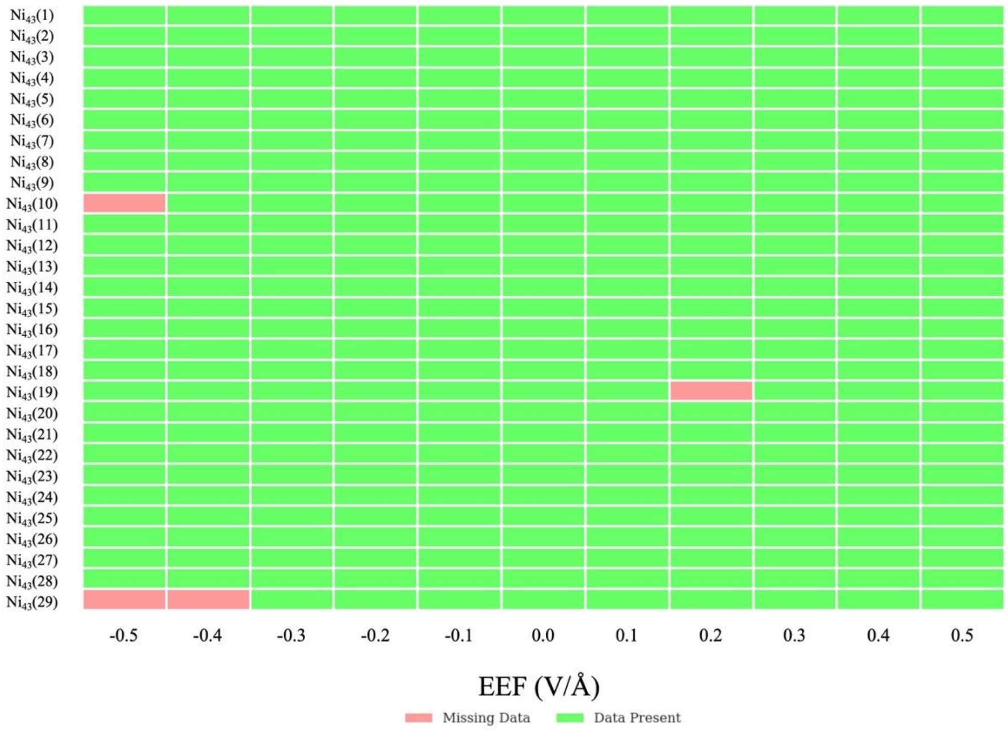

Figure S14. Data availability heatmap for Ni_{43} (0.87 nm to 1.09 nm).

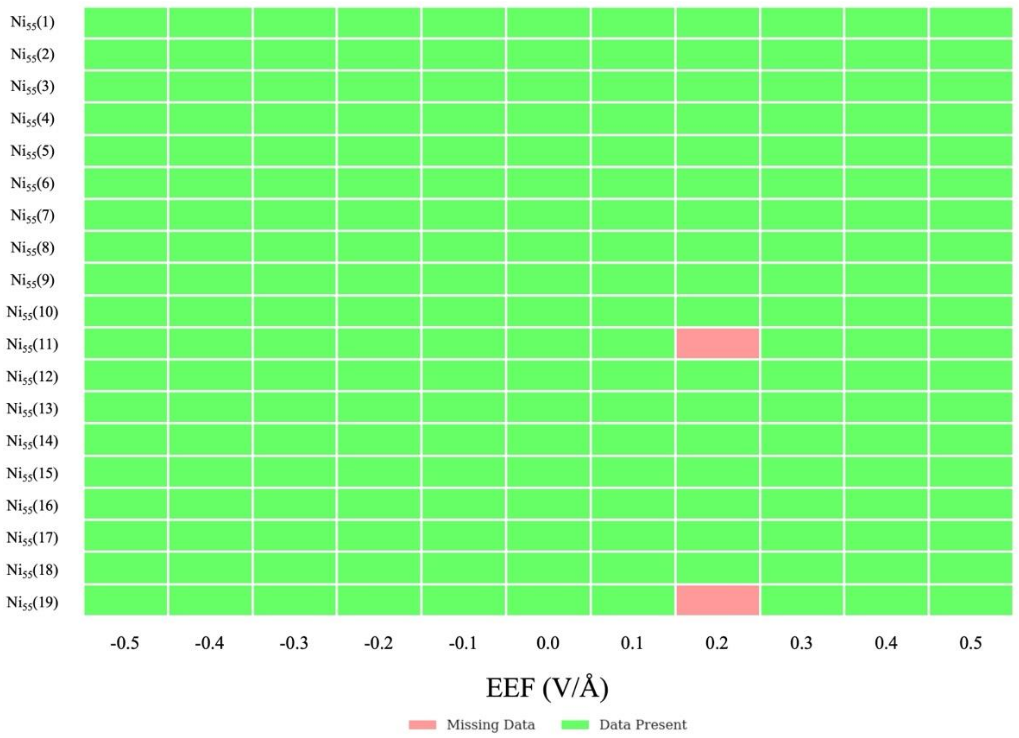

Figure S15. Data availability heatmap for Ni_{55} (0.93 nm to 1.19 nm).

4.3 ML Input Features

Table S4. Input features used for predicting local electric fields.

	Input Features (X)			Prediction (Y)
Model Type	GCN	Cluster Size (nm)	EEF (V/Å)	Local Electric Field (V/Å)
DFT-based	✓	✓	✓	

Table S5. Input Features of two ML approaches for predicting adsorption energy with EEF.

	Input Features (X)					Prediction (Y)
Model Type	GCN	Cluster Size (nm)	Zero-field CO E_{ads} (eV)	EEF (V/Å)	Taylor Expansion (eV)	Adsorption energy in the presence of EEF (eV)
DFT-based ML	✓	✓	✓	✓	---	
Physics Principle Enhanced ML	✓	✓	✓	✓	✓	

4.4 LEF Prediction Learning Curve and Optimized Hyperparameters

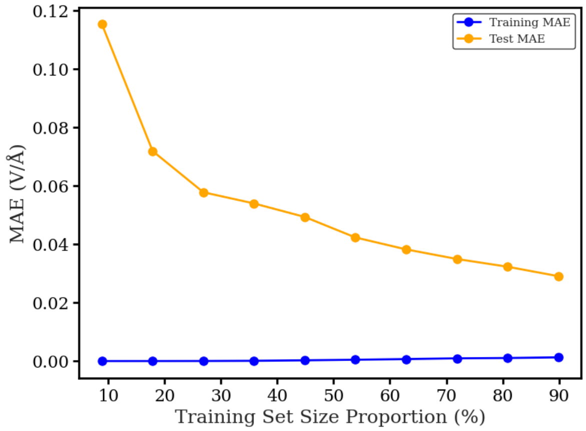

Figure S16. Learning curves depicting training MAE and test MAE for ML in predicting LEFs using Gradient Boosting (GB) model.

We evaluated a diverse range of machine learning models to predict LEFs. The ML models included Linear Regression (LR), Polynomial Regression (PR), Ridge Regression (Ridge), K-Nearest Neighbors (KNN), Multilayer Perceptron (MLP), Random Forest (RF), Extra Trees Regressor (ET), Support Vector Regression (SVM), Decision Tree (DT), Gradient Boosting (GB), AdaBoost (AB), and XGBoost (XGB). To ensure optimal performance and reliability, we employed several optimization strategies. We used 10-fold cross-validation to obtain robust performance estimates across different subsets of our data. Hyperparameter optimization was conducted using both grid search and random search methods, allowing us to fine-tune each model's parameters to achieve their best performance. **Tables S6** shown the optimized hyperparameters of ML models for LEF.

Table S6. Optimized hyperparameters and Test MAE of ML models for local electric field (LEF) prediction.

Model	Optimized Hyperparameters	Test MAE (V/Å)
Ridge	α: 0.1	0.1205
k-Nearest Neighbors	n_neighbors: 9, weights: 'distance'	0.0659
Multi-layer Perceptron	hidden_layer_sizes: (50,50), α: 0.01	0.0633
Random Forest	n_estimators: 200, max_depth: None, min_samples_split: 10	0.0527
Extra Trees	n_estimators: 200, max_depth: 10, min_samples_split: 5	0.0547
Support Vector Machine	C: 1, kernel: 'rbf', gamma: 'scale'	0.0612
Decision Tree	max_depth: 10, min_samples_split: 10	0.0696
Gradient Boosting	n_estimators: 200, learning_rate: 0.3, max_depth: 5	0.0311
AdaBoost	n_estimators: 100, learning_rate: 0.1	0.0940
XGBoost	n_estimators: 200, learning_rate: 0.3, max_depth: 5	0.0313
Polynomial Regression	poly_degree: 2, interaction_only: True	0.0578

4.5 Data Selections, Splitting, and Learning Curves for Field-Dependent Adsorption Prediction

Table S7. Data splitting strategies for predicting adsorption energy in the presence of EEF

		EEF (V/Å)				
Selection Rules	Splitting	±0.1	±0.2	±0.3	±0.4	±0.5
EEF=±0.3 V/Å	Training	---	---	✓	---	---
	Validation	---	---	✓	---	---
	Test	✓	✓	✓	✓	✓
EEF=±0.3, ±0.5 V/Å	Training	---	---	✓	---	✓
	Validation	---	---	✓	---	✓
	Test	✓	✓	✓	✓	✓
80% of All	Training	✓	✓	✓	✓	✓
	Validation	✓	✓	✓	✓	✓
	Test	✓	✓	✓	✓	✓

Table S8. Different training data selection and performance for predicting adsorption energy with EEF

Selection Rules	EEF=±0.3 V/Å	EEF=±0.3, ±0.5 V/Å	80% of All
Subset	146	289	NA
Training	87	173	573
Validation	29	58	72
Test	601	486	72
Test MAE / eV (DFT-based ML)	0.0330	0.0222	0.0103
Test MAE / eV (Physics Principle Enhanced ML)	0.0081	0.0050	0.0048
Training Data Preparation Computational Time (hour)	947.77	1579.53	4633.56
Model Training Time (DFT -based ML, hour)	0.005	0.006	0.007
Model Training Time (Enhanced ML, hour)	0.006	0.007	0.008
The Best Model for DFT-based ML	Extra Trees	Extra Trees	Gradient Boosting
The Best Model for Enhanced ML	Polynomial Regression	Polynomial Regression	Polynomial Regression

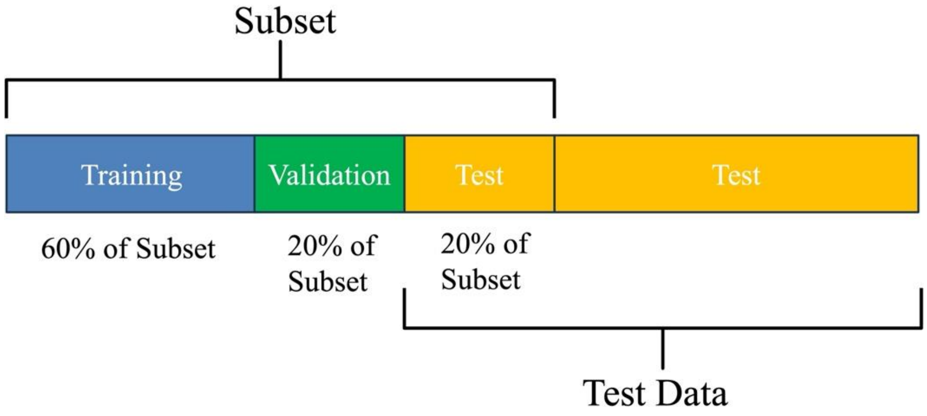

Figure S17. The data splitting method for predicting the adsorption energy in the presence of EEF using DFT-based ML models and physics principal enhanced ML models.

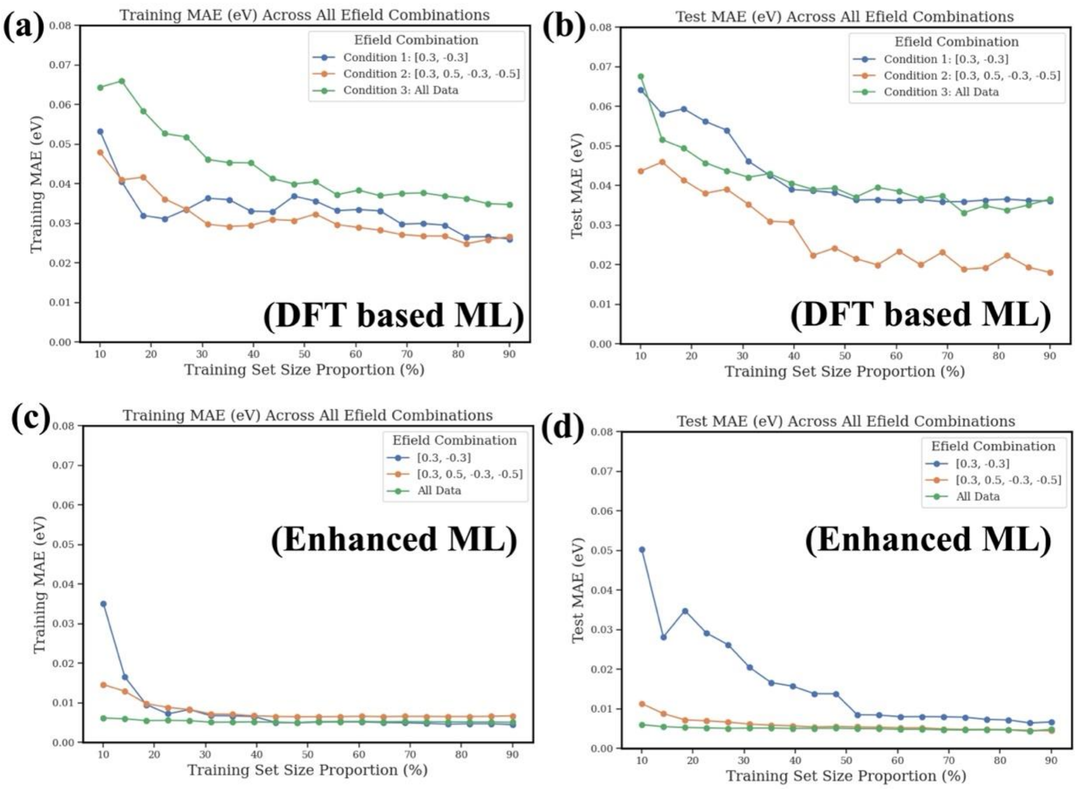

Figure S18. Learning curve for training MAE and test MAE for DFT-based ML and physics principles enhanced ML algorithms when predicting field-dependent adsorption energies. The DFT-based ML approach relies solely on DFT-computed data, while the physics principles enhanced ML approach integrates a first-order Taylor expansion ($E_{ad}(\vec{F}_{local}) = E_{ad} - \Delta\vec{\mu} \cdot \vec{F}_{local}$, where E_{ad} represents the adsorption energy without EEFs, $\Delta\vec{\mu}$ denotes the effective dipole moment, $\vec{F}_{local}$ is the local electric field predicted from our ML model) as a training input, enabling it to achieve comparable accuracy with fewer training data compared to DFT-based ML model.

4.6 Taylor expansion for Field-Dependent Adsorption

$$E_{ad}\left(\vec{F}_{local}\right) = E_{ad} - \Delta\vec{\mu} \cdot \vec{F}_{local} \quad (1)$$

where E_{ad} represents the adsorption energy without EEFs, $\Delta\vec{\mu}$ denotes the effective dipole moment (approximately -0.10 e·Å, reflecting their charge separation of the species in gas phase, the estimation is showing in **Figure S19a**).

Using the Taylor expansion (**Equation (1)**), we estimated field-dependent CO adsorption energies based on DFT-calculated zero-field energies, ML-predicted LEFs, and the effective dipole moment of CO (-0.10 e·Å). Only using physics principles, we achieved a MAE of 0.06 eV, within the DFT error of 0.10 eV[4] (**Figure S19b**).

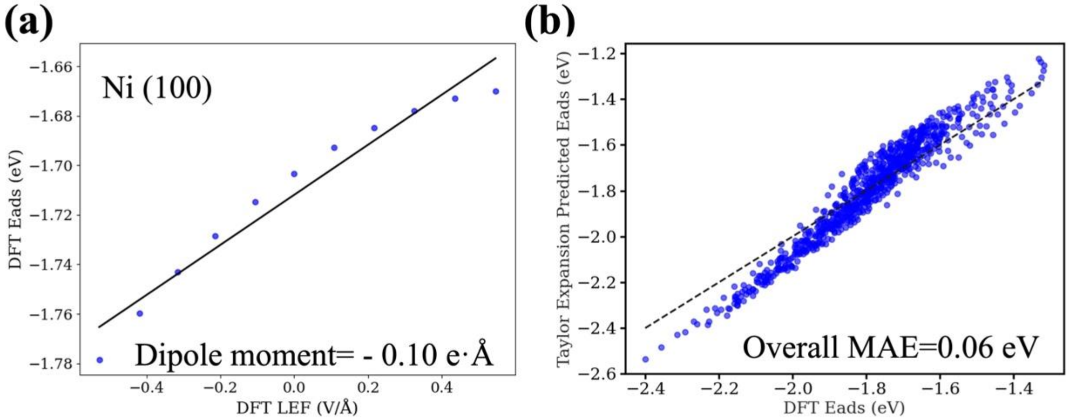

Figure S19. (a) Estimated effective dipole moment via the assumption that LEF has the same magnitude as EEF over the top site over Ni(100). (b) Performance for all the dataset (717 data points) between the first order Taylor expansion in predicting field-dependent CO adsorption energies and DFT-calculated ones.

4.7 Field-Dependent Adsorption Prediction and Optimized Hyperparameters

We evaluated a diverse range of machine learning models to predict field-dependent adsorption energies. The ML models included Linear Regression (LR), Polynomial Regression (PR), Ridge Regression (Ridge), K-Nearest Neighbors (KNN), Multilayer Perceptron (MLP), Random Forest (RF), Extra Trees Regressor (ET), Support Vector Regression (SVM), Decision Tree (DT), Gradient Boosting (GB), AdaBoost (AB), and XGBoost (XGB). To ensure optimal performance and reliability, we employed several optimization strategies. We used 10-fold cross-validation to obtain robust performance estimates across different subsets of our data. Hyperparameter optimization was conducted using both grid search and random search methods, allowing us to fine-tune each model's parameters to achieve their best performance. **Tables S9–S11 and S12–S14** present the optimized hyperparameters for the DFT-based and physics principles enhanced ML models, respectively, used in predicting field-dependent CO adsorption energies.

Table S9. Optimized hyperparameters and test MAE for DFT-based ML models predicting field-dependent CO adsorption energies at EEF = ±0.3 V/Å.

Model	Optimized Hyperparameters	Test MAE (eV)
Ridge	α: 1	0.0444
Lasso	α: 0.1	0.1190
Elastic Net	α: 0.1, l1_ratio: 0.1	0.0463
k-Nearest Neighbors	n_neighbors: 3, weights: 'distance'	0.0565
Multi-layer Perceptron	hidden_layer_sizes: (50,), α: 0.01	0.1491
Random Forest	n_estimators: 50, max_depth: 10, min_samples_split: 2	0.0555
Extra Trees	n_estimators:200, max_depth: 10, min_samples_split: 2	0.0330
Support Vector Machine	C: 10, kernel: 'rbf', gamma: 'auto'	0.0589
Decision Tree	max_depth: None, min_samples_split: 2	0.0562
Gradient Boosting	n_estimators: 200, learning_rate: 2, max_depth: 10	0.0896
AdaBoost	n_estimators: 100, learning_rate: 1	0.0591
XGBoost	n_estimators: 50, learning_rate: 0.1, max_depth: 3	0.0547
Polynomial Regression	poly_degree: 2, interaction_only: True	0.0331

Table S10. Optimized hyperparameters and test MAE for DFT-based ML models predicting field-dependent CO adsorption energies at EEF = ±0.3, ±0.5 V/Å

Model	Optimized Hyperparameters	MAE (eV)
Ridge	α: 1.0	0.0474
Lasso	α: 0.1	0.1101
Elastic Net	α: 0.1, l1_ratio: 0.1	0.0493
k-Nearest Neighbors	n_neighbors: 3, weights: 'distance'	0.0461
Multi-layer Perceptron	hidden_layer_sizes: (50,50), α: 0.0001	0.1160
Random Forest	n_estimators: 100, max_depth: 10, min_samples_split: 2	0.0499
Extra Trees	n_estimators: 100, max_depth: None, min_samples_split: 2	0.0222
Support Vector Machine	C: 1, kernel: 'rbf', gamma: 'scale'	0.0430
Decision Tree	max_depth: 10, min_samples_split: 2	0.0499
Gradient Boosting	n_estimators: 200, learning_rate: 0.3, max_depth: 3	0.0462
AdaBoost	n_estimators: 200, learning_rate: 1.0	0.0544
XGBoost	n_estimators: 100, learning_rate: 0.3, max_depth: 5	0.0878
Polynomial Regression	poly_degree: 2, interaction_only: True	0.0378

Table S11. Optimized hyperparameters and test MAE for DFT-based ML models predicting field-dependent CO adsorption energies sing 80% of the entire dataset.

Model	Optimized Hyperparameters	MAE (eV)
Ridge	α: 1.0	0.0422
Lasso	α: 0.1	0.1203
Elastic Net	α: 0.1, l1_ratio: 0.1	0.0414
k-Nearest Neighbors	n_neighbors: 3, weights: 'distance'	0.0197
Multi-layer Perceptron	hidden_layer_sizes: (50,50), α: 0.01	0.0409
Random Forest	n_estimators: 200, max_depth: None, min_samples_split: 2	0.0165
Extra Trees	n_estimators: 200, max_depth: 20, min_samples_split: 2	0.0152
Support Vector Machine	C: 1, kernel: 'rbf', gamma: 'scale'	0.0301
Decision Tree	max_depth: 10, min_samples_split: 2	0.0233
Gradient Boosting	n_estimators: 200, learning_rate: 0.1, max_depth: 5	0.0103
AdaBoost	n_estimators: 50, learning_rate: 1.0	0.0375
XGBoost	n_estimators: 200, learning_rate: 0.1, max_depth: 5	0.0107
Polynomial Regression	poly_degree: 2, interaction_only: True	0.0305

Table S12. Optimized hyperparameters and test MAE for physical principle enhanced ML models predicting field-dependent CO adsorption energies at EEF = ±0.3 V/Å.

Model	Optimized Hyperparameters	Test MAE (eV)
Ridge	α: 0.1	0.0111
Lasso	α: 0.1	0.0854
Elastic Net	α: 0.1, l1_ratio: 0.1	0.0230
k-Nearest Neighbors	n_neighbors: 3, weights: 'distance'	0.0419
Multi-layer Perceptron	hidden_layer_sizes: (50,50), α: 0.01	0.2884
Random Forest	n_estimators: 200, max_depth: None, min_samples_split: 2	0.0242
Extra Trees	n_estimators:200, max_depth: 10, min_samples_split: 2	0.0171
Support Vector Machine	C: 10, kernel: 'rbf', gamma: 'auto'	0.0513
Decision Tree	max_depth: None, min_samples_split: 2	0.0330
Gradient Boosting	n_estimators: 100, learning_rate: 0.1, max_depth: 5	0.0227
AdaBoost	n_estimators: 200, learning_rate: 1.0	0.0289
XGBoost	n_estimators: 100, learning_rate: 0.1, max_depth: 5	0.0197
Polynomial Regression	poly_degree: 2, interaction_only: True	0.0081

Table S13. Optimized hyperparameters and test MAE for physical principle enhanced ML models predicting field-dependent CO adsorption energies at EEF = ±0.3, ±0.5 V/Å.

Model	Optimized Hyperparameters	Test MAE (eV)
Ridge	α: 0.1	0.0108
Lasso	α: 0.1	0.0789
Elastic Net	α: 0.1, l1_ratio: 0.1	0.0241
k-Nearest Neighbors	n_neighbors: 5, weights: 'distance'	0.0354
Multi-layer Perceptron	hidden_layer_sizes: (50,50), α: 0.001	0.1366
Random Forest	n_estimators: 50, max_depth: None, min_samples_split: 2	0.0187
Extra Trees	n_estimators: 200, max_depth: None, min_samples_split: 2	0.0118
Support Vector Machine	C: 10, kernel: 'rbf', gamma: 'auto'	0.0302
Decision Tree	max_depth: None, min_samples_split: 2	0.0256
Gradient Boosting	n_estimators: 200, learning_rate: 0.1, max_depth: 3	0.0124
AdaBoost	n_estimators: 200, learning_rate: 1.0	0.0243
XGBoost	n_estimators: 200, learning_rate: 0.1, max_depth: 3	0.0125
Polynomial Regression	poly_degree: 2, interaction_only: True	0.0050

Table S14. Optimized hyperparameters and test MAE for physical principle enhanced ML models predicting field-dependent CO adsorption energies sing 80% of the entire dataset.

Model	Optimized Hyperparameters	Test MAE (eV)
Ridge	α: 0.1	0.0108
Lasso	α: 0.1	0.0856
Elastic Net	α: 0.1, l1_ratio: 0.1	0.0186
k-Nearest Neighbors	n_neighbors: 3, weights: 'distance'	0.0126
Multi-layer Perceptron	hidden_layer_sizes: (50,50), α: 0.01	0.0613
Random Forest	n_estimators: 100, max_depth: None, min_samples_split: 2	0.0092
Extra Trees	n_estimators: 200, max_depth: 20, min_samples_split: 2	0.0067
Support Vector Machine	C: 10, kernel: 'rbf', gamma: 'auto'	0.0324
Decision Tree	max_depth: 10, min_samples_split: 2	0.0163
Gradient Boosting	n_estimators: 200, learning_rate: 0.1, max_depth: 3	0.0065
AdaBoost	n_estimators: 200, learning_rate: 1.0	0.0184
XGBoost	n_estimators: 200, learning_rate: 0.3, max_depth: 3	0.0072
Polynomial Regression	poly_degree: 2, interaction_only: True	0.0048

Table S15. R^2 of different models in predicting field-dependent adsorption energies.

Model	R^2
Eads(Equation Predicted) vs DFT calculated Eads(F)	0.8131
Eads(ML without equation) vs DFT calculated Eads(F)	0.9063
Eads(ML with equation) vs DFT calculated Eads(F)	0.9965

4.8 Pearson Correlation Analysis for the Physics-Principles-Enhanced ML Model

To further demonstrate that our physics principles enhanced machine learning (ML) model derives its superior performance from the embedded physical insights, we performed a Pearson correlation analysis on all relevant input features under the applied external electric fields (EEF) of ±0.3, ±0.5. Specifically, we examined the following descriptors: (1) the external electric field (EEF), (2) the generalized coordination number (GCN), (3) the cluster size, (4) the zero-field CO adsorption energy, and (5) the Taylor expansion. The goal was to verify that the physics principles contribute the most critical factor to predict adsorption energies under various EEFs.

We use the standard Pearson correlation coefficient ρ, defined as

$$\rho_{X,Y} = \frac{\sum_{i=1}^{n}(X_i - \overline{X})\,(Y_i - \overline{Y})}{\sqrt{\sum_{i=1}^{n}(X_i - \overline{X})^2}\,\sqrt{\sum_{i=1}^{n}(Y_i - \overline{Y})^2}}$$

where X_i and $Y_i$are values of features X and Y, respectively, across the n data samples, and $\overline{X}(\overline{Y})$ is the mean of X(Y). **Figure S20** summarizes the resulting correlation matrix for the scenarios of EEF = ±0.3, ±0.5 V/Å. Notably, the physics principles (Taylor expansion) display a strong positive correlation with the predictions of field-dependent adsorption energies, confirming that our model's accuracy largely stems from encoding the physics principles. While geometric descriptors like GCN and cluster size still affect the predictions of field-dependent adsorption energies, their correlations are less impacted than the physics principles.

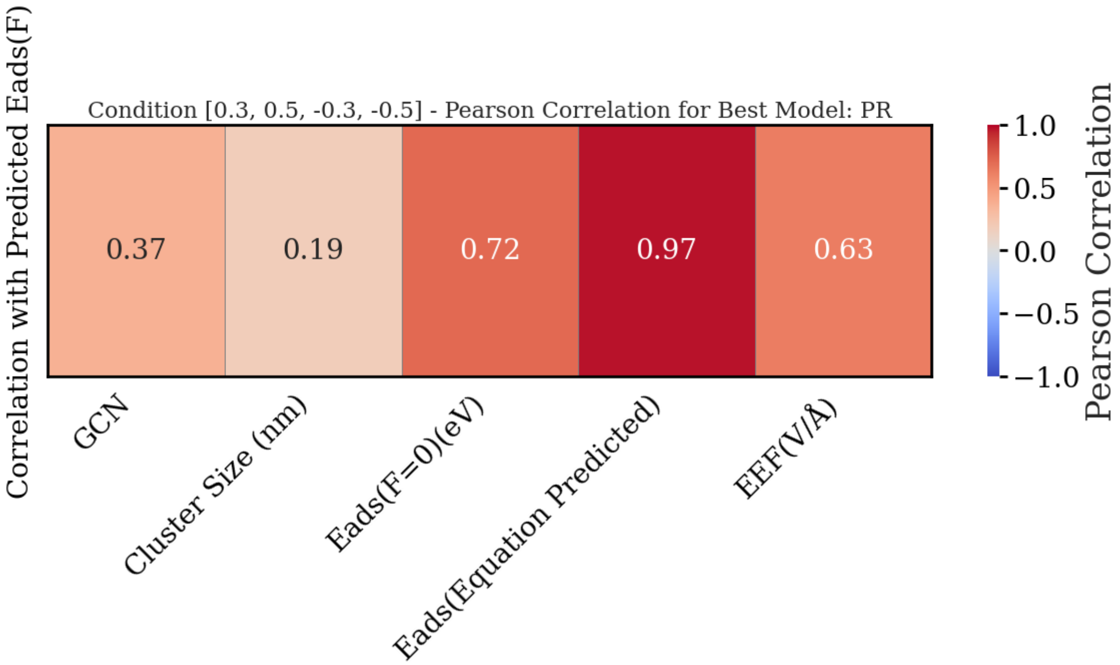

Figure S20. Pearson Correlation between input features for physics principles enhanced ML model for the scenarios of EEF = ±0.3, ±0.5 V/Å.

4.9 Transferability of the ML Models

Table S16. Performance of linear regression for correcting PD-mapped LEFs using VSE for Ir

	Equations	MAE (V/Å)	RSME (V/Å)	R^2
Slab	VSE = -0.1+ 1.04 × PD	0.05	0.06	0.969
Cluster	VSE = +0.01+ 1.09× PD	0.27	0.55	0.946

Note: Here, MAE, RSME and R^2 are based on the difference between Ni-trained model predictions and LEFs calculated by DFT-based VSE for all Ir NPs/slabs data.

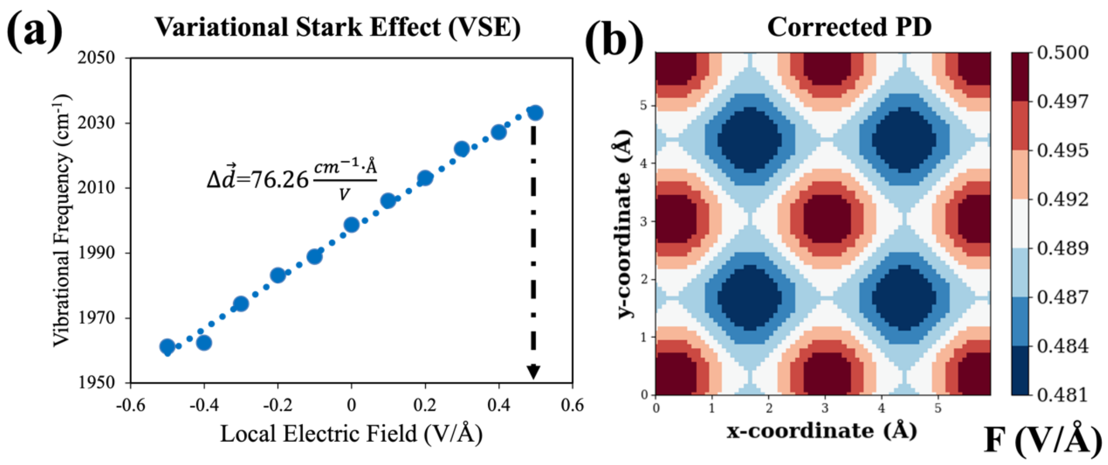

Figure S21. (a) The LEF of the top site of Ir(100) using VSE method. (b) The LEF distribution of the x-y plane of the Ir(100) surface using the PD method. Our implementation of the VSE method for Ir systems is similar to the Ni system and relies on two key assumptions. First, we assumed the LEFs of the top site of Ir(100) are identical to the EEFs. Second, we assumed that the tuning rate (i.e., effective dipole moment of adsorbed CO) remains consistent across different surface environments when bound to a single Ir atom in the top configuration.

References

(1) Brewer, S. H.; Franzen, S. A quantitative theory and computational approach for the vibrational Stark effect. *The Journal of Chemical Physics* **2003**, *119* (2), 851-858. DOI: 10.1063/1.1578471 (acccessed 12/22/2024).

(2) Wright, D.; Sangtarash, S.; Mueller, N. S.; Lin, Q.; Sadeghi, H.; Baumberg, J. J. Vibrational Stark Effects: Ionic Influence on Local Fields. *The Journal of Physical Chemistry Letters* **2022**, *13* (22), 4905-4911. DOI: 10.1021/acs.jpclett.2c01048.

(3) Garrett, B. F.; Azuri, I.; Kronik, L.; Chelikowsky, J. R. Real-space pseudopotential method for computing the vibrational Stark effect. *The Journal of Chemical Physics* **2016**, *145* (17). DOI: 10.1063/1.4965918 (acccessed 12/22/2024).

(4) Wellendorff, J.; Silbaugh, T. L.; Garcia-Pintos, D.; Nørskov, J. K.; Bligaard, T.; Studt, F.; Campbell, C. T. A benchmark database for adsorption bond energies to transition metal surfaces and comparison to selected DFT functionals. *Surface Science* **2015**, *640*, 36-44. DOI: 10.1016/j.susc.2015.03.023.
